# Rab geranylgeranyl transferase activity is required for proper sterol biosynthesis in *Arabidopsis thaliana*

**DOI:** 10.1093/pcp/pcaf166

**Published:** 2025-12-10

**Authors:** Małgorzata Gutkowska, Marta Zajbt-Łuczniewska, Daniel Buszewicz, Anna Anielska-Mazur, Agata Lipko, Cezary Pączkowski, Grzegorz Spólnik, Maciej Sojka, Radosław Jaźwiec, Emilia Samborowska, Ewa Swiezewska, Marta Hoffman

**Affiliations:** Department of Biochemistry and Microbiology, Institute of Biology, Warsaw University of Life Sciences, ul. Nowoursynowska 159, bldg. 37, 02-776 Warsaw, Poland; Institute of Biochemistry and Biophysics, Polish Academy of Sciences, ul. Pawińskiego 5a, 02-106 Warsaw, Poland; Institute of Biochemistry and Biophysics, Polish Academy of Sciences, ul. Pawińskiego 5a, 02-106 Warsaw, Poland; Institute of Biochemistry and Biophysics, Polish Academy of Sciences, ul. Pawińskiego 5a, 02-106 Warsaw, Poland; Institute of Biocybernetics and Biomedical Engineering, Polish Academy of Sciences, ul. Ks. Trojdena 4, 02-109 Warsaw, Poland; Department of Plant Biochemistry, Institute of Biochemistry, Faculty of Biology, University of Warsaw, ul. Miecznikowa 1, 02-096 Warsaw, Poland; Institute of Organic Chemistry, Polish Academy of Sciences, ul. Kasprzaka 44/52, 01-224 Warsaw, Poland; Institute of Organic Chemistry, Polish Academy of Sciences, ul. Kasprzaka 44/52, 01-224 Warsaw, Poland; Institute of Biochemistry and Biophysics, Polish Academy of Sciences, ul. Pawińskiego 5a, 02-106 Warsaw, Poland; Institute of Biochemistry and Biophysics, Polish Academy of Sciences, ul. Pawińskiego 5a, 02-106 Warsaw, Poland; Institute of Biochemistry and Biophysics, Polish Academy of Sciences, ul. Pawińskiego 5a, 02-106 Warsaw, Poland; Institute of Biochemistry and Biophysics, Polish Academy of Sciences, ul. Pawińskiego 5a, 02-106 Warsaw, Poland

**Keywords:** isoprenoid lipids, sterols, dolichols, protein geranylgeranylation, *Arabidopsis*

## Abstract

In all eukaryotic cells, protein prenylation and cytoplasmic isoprenoid biosynthesis pathways leading to sterols, dolichols, and other isoprenoid compounds share a common precursor pool of isopentenyl diphosphate and the isomeric dimethylallyl diphosphate. Despite this, little is known about the interplay between these processes. Here we ask whether perturbation of protein prenylation in plants influences isoprenoid biosynthesis in the endoplasmic reticulum, and in particular if it affects sterol and dolichol biosynthesis. We use an *Arabidopsis thaliana* mutant with defects in the Rab geranylgeranyl transferase as a viable model of protein hypoprenylation, and we show that sterol and dolichol content is significantly elevated in the mutant plants. Also sterol composition is changed: cholesterol content is increased and some atypical sterol pathway intermediates are accumulating. Our results show that plant sterol biosynthesis involves high levels of crosstalk between pathway branches than previously reported and receives regulatory input from protein prenylation pathways.

## Introduction

Protein prenylation is a posttranslational modification in which a 15-carbon farnesyl or 20-carbon geranylgeranyl lipid anchor is being added to specific cysteine residues of a peptide chain. Prenylated proteins are known to be involved in numerous cellular processes, e.g. intracellular transport and signal transduction pathways. In plants, prenylated proteins include Rab GTPases, Rho GTPases, γ-subunits of trimeric G-proteins, and many farnesylated proteins, and they play crucial roles in growth, polarity establishment, plant development, fertility, and reactions to environmental stimuli (reviewed by Running 2014, Turnbull and Hemsley 2017, Hala and Zarsky 2019). The role of prenylated proteins in cell biology is well established but it is often overlooked that the prenyl anchor synthesis overlaps with the prenyl lipid formation pathway. In plants, similar to other Eukaryotes, disruption of isoprenoid biosynthesis in the cytoplasm upstream of geranylgeranyl diphosphate (GGPP) or farnesyl diphosphate (FPP) affects protein prenylation (Randall et al. 1993, Shipton et al. 1995, Rodriguez-Concepcion et al. 1999, Gutkowska et al. 2004). In mammals and yeasts, it is also known that the synthesis of GGPP (or protein geranylgeranylation) influences the flux of metabolites into sterol biosynthesis by regulating the stability of the key enzyme of the pathway, the HMG-CoA reductase (Garza et al. 2009, Leichner et al. 2011, Elsabrouty et al. 2021, Wangeline and Hampton 2021, Faulkner and Jo 2022).

To date, three different protein prenyltransferases have been described in plants (reviewed in Hala and Zarsky 2019) and the existence of a fourth one is highly likely according to genomic analyses (Tateishi et al. 2025). The first two are farnesyltransferase (FTase) and geranylgeranyl transferase I (GGTase I), which recognize a short prenylation sequence at the C-termini of proteins: -CAAX (Marchwicka et al. 2022). FTase in plants modifies diverse proteins such as calmodulin, transcription factors, and most importantly DNAJ chaperones, and the main GGTase I subjects are Rho proteins and γ-subunits of heterotrimeric G-proteins (Hala and Zarsky 2019). The proteins modified by CAAX-prenyltransferases are important yet minor constituents of the plant cell, as the concomitant lack of both FTase and GGTase I activities in the *Arabidopsis plp* mutant is non-lethal, and plants survive until maturity (Running et al. 2004). The two remaining protein prenyltransferases in plants are Rab geranylgeranyl transferase (GGTase II, named also RGT), which modifies only Rab proteins (regulators of intracellular vesicular transport; Hala et al. 2010), and (putative) GGTase III, whose yeast counterpart modifies specifically the SNARE protein Ykt6 (involved in membrane fusion events; Shirakawa et al. 2020, Tateishi et al. 2025).

Rab proteins are key players in intracellular vesicular traffic in all eukaryotic cells and are also a very populous group of prenylated proteins in the cell. Their geranylgeranylated forms are present on the cytoplasmic face of internal membranes, including the endoplasmic reticulum (ER) membrane. RGT introduces two geranylgeranyl moieties on each Rab protein. The enzyme consists of two subunits: α (RGTA), β (RGTB) and accessory protein called REP (Rab escort protein) (reviewed in Wang and Casey 2016, Marchwicka et al. 2022). Of these, in the *Arabidopsis* genome, the α and β subunits are each encoded by two genes: *RGTA1* and *RGTA2* (possibly a pseudogene), and *RGTB1* and *RGTB2*, while REP is encoded by a single gene (Hala et al. 2010, Shi et al. 2016). Knocking-out of the *REP* gene causes pollen sterility (Gutkowska et al. 2021), but knocking-out of the *RGTB1* gene, encoding for the more abundant isoform of the β subunit, leaves the plants viable though nearly sterile (Hala et al. 2010, Gutkowska et al. 2015, Rojek et al. 2021a, 2021b). Furthermore, in yeast and mammals, the β subunit of RGT is shared by the GGTase III and serves in Ykt6 protein geranylgeranylation without the REP protein (Shirakawa et al. 2020, Tateishi et al. 2025). Because of the high abundance of Rab proteins in the cell, the sporophyte viability of the mutant, and the easily recognizable phenotypes of mature mutant plants (bushy dwarfs), *rgtb1* plants are a convenient model for studying protein hypoprenylation (Hala et al. 2010).

**Figure 1:**
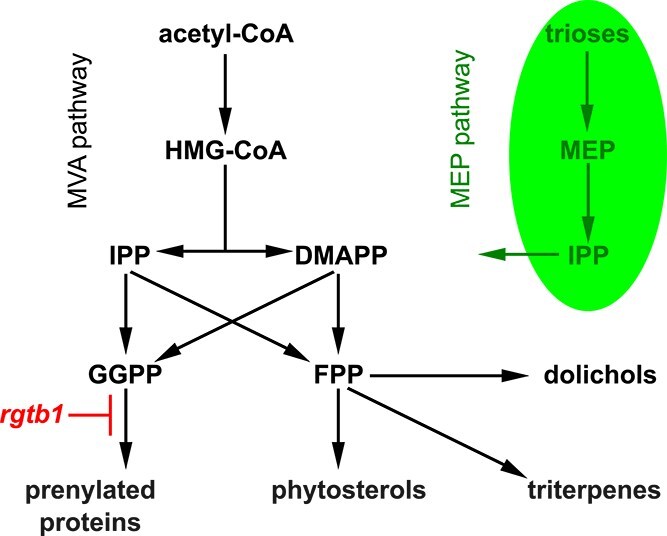
Isoprenoid biosynthesis in plants. Schematic representation of isoprenoid synthesis pathways in plants. Green oval represents a chloroplast and all pathways marked inside it operate in chloroplasts. All black marked pathways operate in the cytoplasm/ER surface/lipid bodies. The position where the *rgtb1* mutation interferes with the isoprenoid pathways is marked on the scheme.

Apart from the protein substrates, all protein prenyltransferases also require the lipid substrates: FPP or GGPP. These moieties are produced in the cells from 5-carbon isoprene precursors, isopentenyl diphosphate (IPP) and its isomer dimethylallyl diphosphate (DMAPP), in reactions catalyzed either by farnesyl diphosphate synthase (FPS) or by geranylgeranyl diphosphate synthase (GGPS) (recently reviewed in Kopcsayova and Vranova 2019, Bergman et al. 2024). Hence, at least partially, GGPP and FPP share the same IPP/DMAPP precursor pool (Fig. 1). In mature, photosynthetically active plants, the situation is further complicated by the existence of two parallel pathways producing IPP and DMAPP (reviewed in Hemmerlin et al. 2012, Bergman et al. 2024). The first pathway (methylerythritol phosphate pathway, MEP) operates only in plastids and utilizes phosphorylated sugar precursors derived from the Calvin-Benson cycle (Hemmerlin et al. 2012, Rodriguez-Concepcion and Boronat 2015, Wang et al. 2023). The MEP pathway strongly depends on light and produces plastidial isoprenoids, including photosynthetic pigments (Rodriguez-Concepcion and Boronat 2015, Ruiz-Sola et al. 2016b). The second pathway producing IPP and DMAPP (mevalonate pathway, MVA) operates in the cytoplasm of all eukaryotic cells and starts with acetyl-CoA (Hemmerlin et al. 2012, Rodriguez-Concepcion and Boronat 2015). The main enzyme controlling the flow of intermediates through the MVA pathway is 3-hydroxy-3-methyl-glutaryl-CoA reductase (HMGR). This enzyme is regulated on multiple levels: transcriptional, posttranslational, allosteric, and degradative (Erffelinck and Goossens 2018).

FPP, which is synthesized in cytoplasm by FPS1S and FPS2 enzymes (Keim et al. 2012), serves as a substrate for several key pathways: it can be converted to sterols in a series of reactions starting from head-to-head condensation of two FPP molecules that forms squalene, or it can be elongated by the addition of further IPP moieties to form the non-cyclic isoprenoid chain of di-*trans*-all-*cis* dolichol, which serves in the ER as carrier of oligosaccharide chains for protein glycosylation, or, finally, FPP may also be used for protein farnesylation (Fig. 1). The cytoplasmic pool of GGPP serves mainly for protein geranylgeranylation and possibly also for the synthesis of the ubiquinone side chain, while the chloroplastic GGPP pool—for the synthesis of photosynthetic pigments, such as carotenoids, chlorophylls, polyprenols, plastoquinone, and tocopherols, as well as secondary metabolites (Ruiz-Sola et al. 2016a, 2016b, Bergman et al. 2024).

In this work, we investigate the possibility that the protein geranylgeranylation pathway influences the biosynthesis of isoprenoids (sterols and/or dolichols) in plants. To study this, we take advantage of the *Arabidopsis thaliana rgtb1* mutant, which is deficient in protein prenylation, but viable (Hala et al. 2010), and we pose the question whether the hypoprenylation displayed by this mutant has any influence on the isoprenoid biosynthesis pathway or cellular processes dependent on sterols and dolichols.

## Results

### Sterol and dolichol levels are increased in *rgtb1* mutant plants

Apart from serving as substrates for protein prenylation, short-chain prenyl diphosphates, FPP and GGPP, are also metabolic precursors for the biosynthesis of two major classes of primary end products of the cytoplasmic MVA pathway: sterols (cyclic isoprenoids) and dolichols (linear, long-chain isoprenoids). Our first step was to analyze the amount of these compounds in *rgtb1–1* and *rgtb1–2* mutants of *Arabidopsis*.

**Figure 2:**
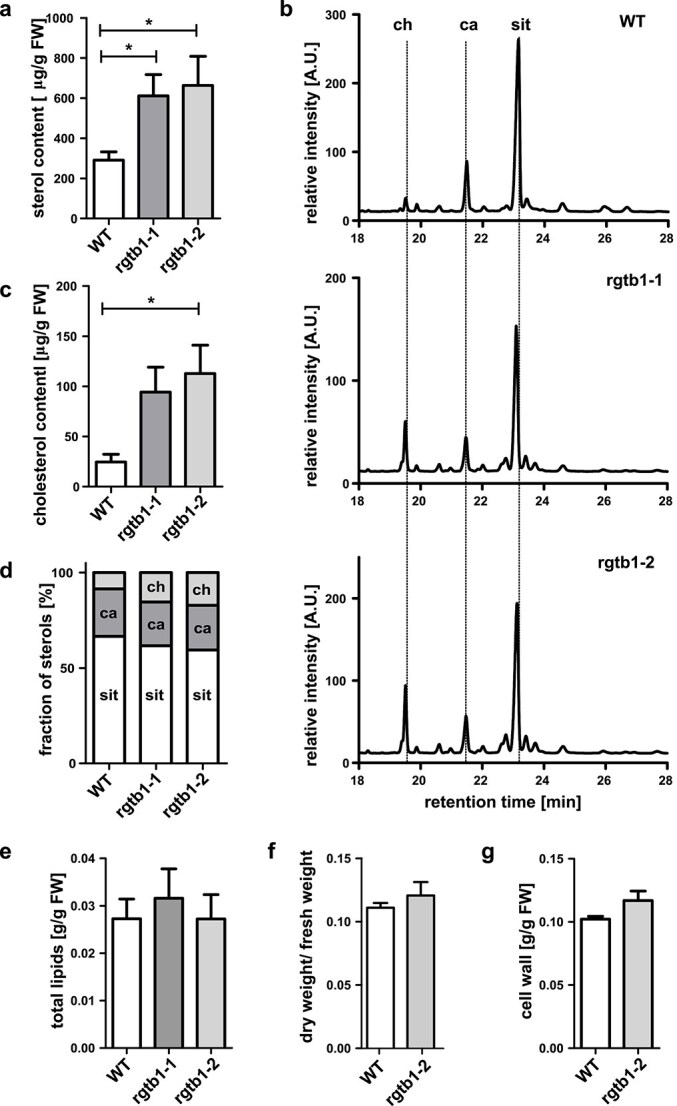
Sterol content and composition in *Arabidopsis rgtb1* and WT plants. Neutral lipids were isolated from whole mature plants grown in soil and fractionated as described in section Materials and Methods. Sterol-containing fractions were analyzed by GC-FID against the internal standard of cholestanol, which was added at the beginning of the extraction procedure, and external standards of sitosterol, campesterol, stigmasterol, and cholesterol. (a) Total amount of sterols per one gram of fresh weight of plant tissue. (b) representative chromatograms of sterol separation by GC-FID. Dotted lines show the retention times of external standards, ch—cholesterol; ca—campesterol, sit—sitosterol. (c) Amount of cholesterol per one gram of fresh weight of plant tissue. (d) Relative amount of the most populated sterols in the analyzed samples: sitosterol (sit—white bar), campesterol (ca—dark grey bar), cholesterol (ch—light grey bar). (e) Quantification of the total lipid weight in the in *rgtb1*mutants versus WT plants. (f) Quantification of the water content in *rgtb1–2* mutant *versus* WT plants, as proportion of dry weight to fresh weight of tissue. (g) Quantification of the cell wall fraction weight in *rgtb1–2* mutant versus WT plants. Graphs represent mean ± SEM. At least five biological replicates were performed for each genotype, coming from 2 to 3 plant cultivations. Statistical analysis was performed using GraphPad PRISM 5.0 package by pairwise comparison with Student’s *t*-test against the H_0_ hypothesis that the values for WT and mutant are equal. Statistical significance at *P* = 0.05 is shown by asterisk.

First we studied the sterol and dolichol content in total lipid extracts from whole, 5-week-old, soil-grown plants. Total sterol amounts in our samples were comparable to previously published results (Diener et al. 2000, Schaeffer et al. 2001, Carland et al. 2010, Closa et al. 2010, Darnet et al. 2020) and reached up to 300 *μ*g of total sterols per gram of fresh weight (FW) of tissue (Table 1, Fig. 2). The overall amount of these compounds was increased over two-fold in *rgtb1* mutants in comparison to WT (Table 1, Fig. 2a). The most abundant sterols in our samples were sitosterol, campesterol, and cholesterol (Fig. 2b). The proportion of campesterol in total main sterols was very similar in samples from WT and mutant plants, but the proportion of cholesterol was increased in *rgtb1* almost two-fold, from 8.5% to 16%, at the expense of sitosterol (Fig. 2b and d), meaning that cholesterol levels reached 100 *μ*g/g FW in the mutants, compared with 25 *μ*g/g FW in WT plants (Fig. 2c). Comparable levels of cholesterol in mature WT plants were found in (Diener et al. 2000). The amount of stigmasterol, often reported in *Arabidopsis* as more abundant than cholesterol, was relatively low in our samples and reached no more than several *μ*g/g FW in WT plants (Table 1).

**Table 1:** Content of sterols, their selected precursors, and other selected triterpenoids in WT and *rgtb1* plants.

**Name**		**Compound content [*μ*g/g FW]** **Mean ± SEM**		
	***m/z***	**WT**	***rgtb1–1***	***rgtb1–2***
Sitosterol	414	193.7 ± 41.7	301.8 ± 97.7	280.7 ± 34.9
Campesterol	400	72.3 ± 11.0	140.0 ± 24.2	155.0 ± 33.6
Cholesterol	386	24.8 ± 7.6	94.4 ± 24.8	112.8 ± 28.3
Stigmasterol	412	3.5 ± 1.0	12.7 ± 4.0	10.9 ± 1.4
Squalene	410	2.1 ± 0.6	2.4 ± 0.6	1.8 ± 0.5
Cycloartanol	428	5.5 ± 1.4	19.1 ± 0.1	18.8 ± 2.9
Cycloartenol	426	17.7 ± 3.6	57.6 ± 0.8	55.5 ± 9.0
24-Methylenecycloartanol	440	n.d.	1.0 ± 0.1	1.1 ± 0.2
Dihydro-t-MAS (4,4-dimethyl-cholest-8-en-3β-ol)	414	6.4 ± 2.1	27.6 ± 5.4	34.4 ± 10.6
α-Amyrin	426	8.2 ± 3.2	25.06 ± 7.9	24.11 ± 12.5
Dihydro-ff-MAS (4,4-dimethyl-cholest-8,14-dien-3β-ol)	412	n.m.	n.m.	n.m.
Obtusifoliol	426	n.m.	n.m.	n.m.
Iso-fucosterol	412	n.m.	n.m.	n.m.
24-Methylene cholesterol	398	n.m.	n.m.	n.m.
14-Methylergosta-8,22-dien-3-ol	412	n.m.	n.m.	n.m.
5α-Stigmast-9(11)-en-3β-ol	414	n.m.	n.m.	n.m.
4-Methyl-stigmasta-4,22-dien-3-one	424	n.m.	n.m.	n.m.
β-Amyrin	426	n.m.	n.m.	n.m.
Ursa-9,12-dien-3-ol	424	n.m.	n.m.	n.m.

Content of selected isoprenoid compounds in WT and *rgtb1* mature plants was measured and quantified as described in section Materials and Methods. Data represent a mean ± SEM of at least three biological replicates for each of the compounds. n.m. non-measured compounds were detected, but were impossible to quantify, because chromatographic peaks overlapped.

We were surprised by the high increase in total sterol levels, so we compared the total lipid content in the mutants, but the ratio of total lipids per FW was similar in WT and mutant plants (Fig. 2e). Also we measured the ratio of dry weight to FW for WT and *rgtb1* plants, which reflects a difference in water content but we found no significant differences (Fig. 2f). Finally, we looked at the ratio of cell wall components to FW to see if the smaller cell size of the mutants would result in a general increase in cell wall components, but the ratio was similar for WT and *rgtb1* plants (Fig. 2g). We conclude that the observed values indeed reflect an increase of sterol pool, not just a physiological difference related to the dwarfism of the studied *rgtb1* plants.

**Figure 3:**
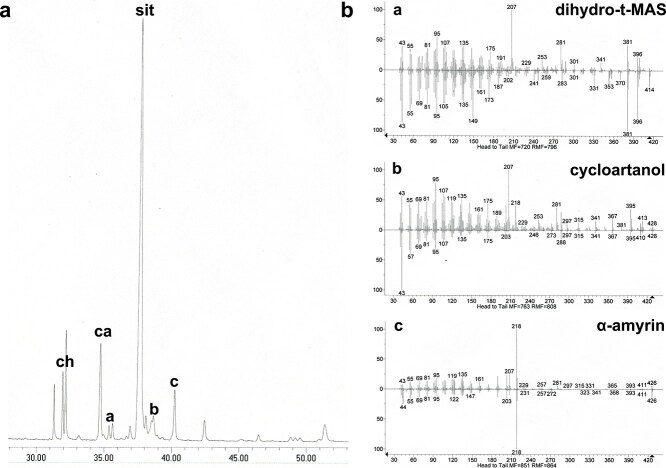
Sterol precursors and selected triterpenoids in *Arabidopsis rgtb1* mutant plants. Neutral lipids were isolated from whole mature plants grown in soil and fractionated as described in section Materials and Methods. Fractions containing sterol precursor compounds were analyzed by GC–MS. Compounds were identified by comparison of the *m/z* value of the main peak and *m/z* of the fragmentation spectra with the NIST database. Full qualitative data are described in the main text and quantification is presented in Table 1. (a) representative chromatogram of GC–MS analysis of *rgtb1–2* mutant. Major sterol peaks are marked above the peaks as: ch—cholesterol, ca—campesterol, sit—sitosterol. Peaks representing unusual sterol precursors and triterpenoids are marked with the letters a,b,c. (b) Fragmentation spectra and *m/z* values of compounds a, b, and c in comparison to spectra of compounds from the NIST database are shown as examples of the steroid identification procedure. Structural chemical formulas of all relevant compounds are shown in Supplementary Fig. S1.

In order to see if the increase in total sterol content and in the relative abundance of cholesterol is accompanied by changes in the content of cyclic sterol precursors, we used GC–MS to analyze lipid fractions enriched in the slightly less polar pathway intermediates. Apart from sitosterol, campesterol, and cholesterol, there were other peaks visible on the chromatograms (Fig. 3a). For the MS fragmentation spectra obtained from this analysis, we performed comparisons with the NIST spectra catalogue which allowed us to identify the following sterol precursors (Table 1, Fig. 3). Some of these compounds were observed only in trace amounts, or in some runs yielded overlapping chromatographic peaks, and hence were difficult to quantify. In Fig. 3a, we show a representative chromatogram from WT plant analysis and MS identification of two atypical compounds, cycloartanol (Fig. 3b compound b) and dihydro-t-MAS (Fig. 3b, compound a). For many of these sterol precursors, we detected an increase in content in *rgtb1* plants in comparison to WT plants (Table 1). Chemical structures of the most interesting compounds that we identified and quantified are shown on Supplementary Fig. S1.

The dihydro-t-MAS compound seemed particularly worthy of attention because its increase was substantial: from 6 *μ*g/g FW in WT plants to 30 *μ*g/g FW in *rgtb1* mutants. This sterol precursor is a known intermediate in the vertebrate’s Kandutsch-Russell pathway of cholesterol biosynthesis (Mazein et al. 2013, Meng et al. 2018) but has never been, to our knowledge, reported in plants. To verify the presence of this novel compound in *Arabidopsis*, we performed Ultra Performance Liquid Chromatography-Mass Spectrometry-Multiple Reaction Monitoring (UPLC-MS-MRM) for an authentic standard of dihydro-t-MAS alongside our own WT and *rgtb1* samples (Supplementary Fig. S2). The characteristic fragmentation ions of *m/z* 285.36, 177.2, and 95.12 were present in all samples. We also detected and verified (again using an authentic standard) another compound from the Kandutsch-Russell pathway, upstream of dihydro-t-MAS, i.e. dihydro-ff-MAS (Supplementary Fig. S2).

**Figure 4:**
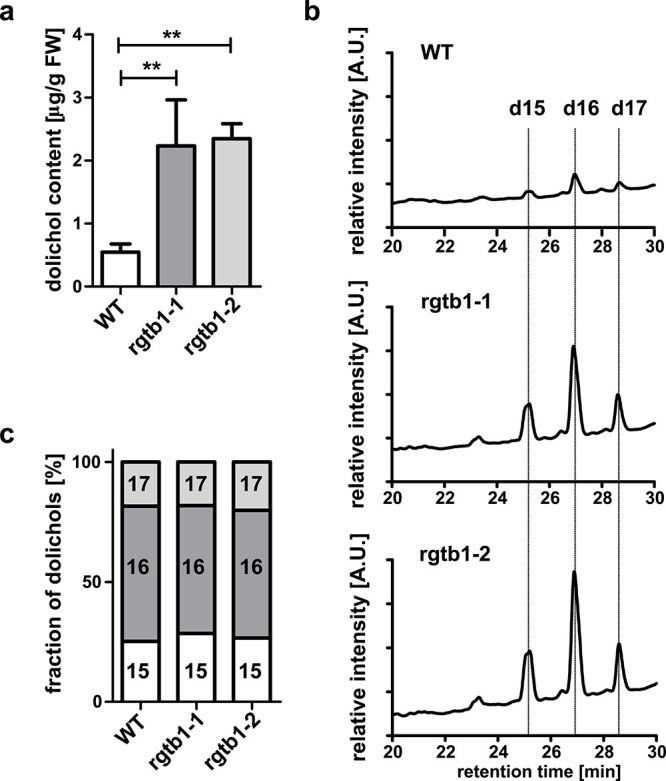
Dolichol content and composition in *Arabidopsis rgtb1* and WT plants. Neutral lipids were isolated from whole mature plants grown in soil and fractionated as described in section Materials and Methods. Dolichol-containing fractions were analyzed by HPLC-UV at 210 nm against the internal standard of prenol-14 or prenol-19, which was added at the beginning of the extraction procedure, and external standards of a prenol 9–25 mixture and a dolichol 17–21 mixture. (a) Total amount of dolichols 15–17 (C75-C80-C85) per one gram of fresh weight of plant tissue. (b) Relative amount of the most populated dolichols in the analyzed samples: dolichol-15 (15—white bar), dolichol-16 (16—dark grey bar), dolichol-17 (17—light grey bar). (c) Representative chromatograms of dolichol separation by HPLC-UV. Dotted lines show the retention times of external standards. Graphs represent mean ± SEM. At least three biological replicates were performed for each genotype, coming from an independent plant cultivation each. Statistical analysis was performed using GraphPad PRISM 5.0 package by pairwise comparison with Student’s *t*-test against the H_0_ hypothesis that the values for WT and mutant are equal. Statistical significance at *P* = 0.01 is shown by two asterisks.

We then assayed the low-polarity fractions of our extracts using HPLC-UV on C18 reversed-phase resin by comparison to external standards of dolichols of known chain lengths and by use of an internal standard (polyprenol-14 or polyprenol-19). This analysis allowed us to identify and quantify dolichols. We found that the quantity of these compounds reached 0.6 *μ*g/g of FW in WT plants (as reported previously, Gawarecka et al. 2022) and increased about five-fold in the mutant plants (Fig. 4a and b), but the composition remained the same (Fig. 4c). The most abundant dolichols in both WT and *rgtb1* plants are built of 15–17 isoprene units with dolichol-16 (80-carbon molecule) dominating.

Finally, the non-polar fractions were studied by HPLC-UV on C18 reversed-phase resin and additionally on GC–MS (Table 1). In these fractions, we detected the linear sterol precursor squalene, whose content was unchanged between WT and *rgtb1* samples (remained on a level of about 2 *μ*g/g FW, similar to previous reports by Rasbery et al. (2007) and Laranjeira et al. (2015) using both these methods. Additionally, by GC–MS, we identified some non-steroid cyclic triterpenes: α-amyrin (Fig. 3a and b compound c; Table 1), β-amyrin, and ursenol, that were found previously in *Arabidopsis* (Husselstein-Muller et al. 2001, Shibuya et al. 2009, Buschhaus and Jetter 2012). These triterpenoids were present in increased amounts in the *rgtb1* mutants compared with WT plants. We were unable to detect lanosterol, a known cyclic precursor of the cholesterol pathway in animals, in any of the samples. This was interesting because dihydro-t-MAS and dihydro-ff-MAS, which we detected in measurable amounts, serve in animal cells as intermediate metabolites of the cholesterol biosynthesis pathway, between lanosterol and cholesterol (chemical structures are shown on Supplementary Fig. S1). To understand this situation better, we studied *A. thaliana* mutants carrying T-DNA insertions in the putative lanosterol synthase gene *LAS1 (At3g450130)* (Supplementary Fig. S3a), but found no growth phenotype of the mature rosettes grown in soil (Supplementary Fig. S3b) nor any differences in the sterol composition (Supplementary Fig. S3c and d). The mature rosettes of *las1–1* and *las1–2* lines did not show any phenotypic difference from WT plants (Supplementary Fig. S3b) and neither control nor *las1* lines contained any lanosterol. What is more, the segregation analysis of these two *LAS1* alleles is Mendelian, meaning that the putative lack of lanosterol does not influence the plant fertility (Supplementary Fig. S3).

Altogether, these results suggest that in the *rgtb1* mutants, there is an increase in the flux of metabolites through the biosynthetic branches leading to sterols and dolichols.

### The transcription of genes encoding enzymes of the MVA pathway and downstream isoprenoid biosynthesis pathways is altered in *rgtb1* plants

**Figure 5:**
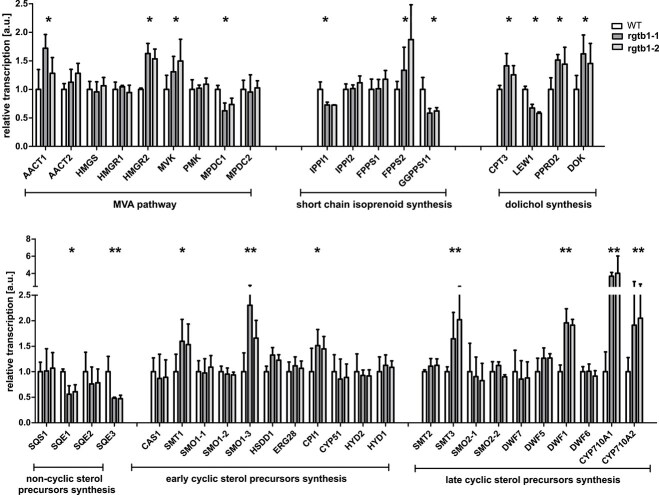
Relative transcription of the genes from the MVA pathway, dolichol biosynthesis pathway and sterol biosynthesis pathway in *Arabidopsis rgtb1* and WT plants. Transcription level of the selected genes was analyzed by RT-qPCR, as described in Material and Methods, for mature *rgtb1* and WT plants grown in soil. Each experiment is a mean of three biological samples, each performed in technical duplicates. Obtained values were normalized to WT values. Bars represent mean ± SD. WT—white bars, *rgtb1–1*—dark grey bars, *rgtb1–2*—light grey bars. Asterisks denote genes with transcription changes of at least 30% in comparison to WT, double asterisks denote genes with transcription changes at least two times in comparison to WT. Full names of the genes, corresponding enzymatic activities, EC numbers and genetic loci are presented in Table 2.

Sterols and dolichols are synthesized from five-carbon isoprene units that under standard conditions in mature plants are derived prevalently from the cytoplasmic MVA pathway (Lipko et al. 2023; see Fig. 1). We studied the transcription of genes coding for enzymes of the MVA pathway and of downstream isoprenoid synthesis steps by using RT-qPCR (names of the genes, corresponding enzymatic activities together with EC number and genetic loci are listed in Table 2; results shown in Fig. 5). To obtain a full picture of the changes occurring in isoprenoid biosynthesis, we also analyzed genes coding for MEP pathway and carotenoid biosynthesis enzymes (listed in Supplementary Table S1; results in Supplementary Fig. S4). We compared transcription levels in mature 5-week old plants of the WT and *rgtb1* genotypes.

**Table 2:** MVA, dolichol, and sterol biosynthesis in plants.

**Gene name**	**Enzymatic activity**	**EC number**	***Arabidopsis* genetic locus number**
**MVA pathway**			
AACT1	Acetyl-CoA C-acetyltransferase	EC 2.3.1.9	At5g47720
AACT2			At5g48230
HMGS	3-Hydroxy-3-methylglutaryl-CoA synthase	EC 2.3.3.10	At4g11820
HMGR1	3-Hydroxy-3-methylglutaryl-CoA reductase	EC 1.1.1.88	At1g76490
HMGR2			At2g17370
MVK	Mevalonate kinase	EC 2.7.1.36	At5g27450
PMK	Phosphomevalonate kinase	*EC 2.7.4.2*	At1g31910
DPMC1	Diphospho-MVA decarboxylase	*EC* 4.1.1.33	At2g38700
DPMC2			At3g54250
**Short chain isoprenoid synthesis**			
IPPI1	Isopentenyl diphosphate isomerase	EC 5.3. 3.2	At5g16440
IPPI2			At3g02780
FPS1	Farnesyl diphosphate synthase	EC 2.5.1.10	At5g47770
FPS2			At4g17190
GGPS11	Geranylgeranyl dipshosphate synthase	EC 2.5.1.29	At4g36810
**Dolichol synthesis**			
CPT3	*cis*-prenyl transferase	EC 2.5.1.87	At2g17570
LEW1	*cis*-prenyl transferase accessory protein	-	At1g11755
PPRD2	Polyprenol reductase	EC 1.3.1.94	At2g16530
DOK	Dolichol kinase	EC 2.7.1.108	At3g45040
**Non-cyclic sterol precursors synthesis**			
SQS1	Squalene synthase	EC 2.5.1.21	At4g34640
SQE1	Squalene epoxidase	EC 1.14.14.17	At1g58440
SQE2			At2g22830
SQE3			At4g37760
**Early cyclic sterol precursors synthesis**			
CAS1	Cycloartenol synthase	EC 5.4.99.8	At2g07050
SMT1	Cycloartenol-C-24- methyltransferase	*EC 2.1.1.41*	At5g13710
SMO1–1	4,4-Dimethylsterol monooxygenase	EC 1.14.18.9	At4g12110
SMO1–2			At4g22756
SMO1–3			At4g22755
HSDD1	Β-Hydroxysteroid dehydrogenase	*EC* 1.1.1.51	At5g50600
ERG28	Scaffolding protein for SMO complex	-	At1g10030
CPI	Cyclopropylsterol isomerase	EC 5.5.1.9	At5g50375
CYP51	Sterol 14-demethylase	EC 1.14.14.154	At1g11680
HYD2	C-8,7-sterol isomerase	EC 5.3.3.5	At1g20050
HYD1	Sterol C-14 reductase	EC 1.3.1.70	At3g52940
**Late cyclic sterol precursors synthesis**			
SMT2	24-Methylenesterol C-methyltransferase	EC 2.1.1.143	At1g20330
SMT3			
SMO2–1	4-Alpha-monomethylsterol monooxygenase	EC 1.14.18.11	At2g29390
SMO2-2			At1g07420
DWF7	Delta(7)-sterol-C5(6)-desaturase 1	EC 1.14.19.20	At3g02580
DWF5	7-Dehydrocholesterol reductase	EC 1.3.1.21	At1g50430
DWF1	Delta-24-sterol reductase	EC 1.3.1.72	At3g19820
DWF6	Steroid-5-alpha-reductase	EC 1.14.11.23	At1g76090
CYP710A1	Sterol C-22 desaturase	*EC* 1.14.19.41	At2g34500
CYP710A2			At2g34490

Full names of the genes of the MVA, dolichol, and sterol biosynthetic pathways in *Arabidopsis*, the corresponding enzymatic activities by enzyme classification and genetic loci in the *A. thaliana* genome.

In *rgtb1* plants, we observed transcriptional up-regulation of three genes encoding enzymes of the MVA pathway: *AACT* (acetyl-CoA C-acetyltransferase), *MVK* (mevalonate kinase), and *HMGR2* (3-hydroxy-3-methylglutaryl reductase 2). Acetyl-CoA C-acetyltransferase is the enzyme catalyzing the first committed step in the synthesis of isoprenoids that diverts the acetyl-CoA molecule from the fatty acid biosynthesis pathway. Particularly interesting was the significant increase in transcription of *HMGR2*, one of the genes encoding the HMGCo-A reductase, but not in the *HMGR1* gene (Enjuto et al. 1994, Suzuki et al. 2009). HMGR is a rate-limiting enzyme which controls metabolite flux through the whole MVA pathway and it is regulated on the levels of transcription, posttranslational modification, protein activity, and stability in yeasts, animals, and plants (Erffelinck and Goossens 2018). Also the mevalonate kinase, MVK is a regulatory enzyme, allosterically reactive to intra- and extracellular cues (Cho et al. 2022).

The only two genes from the MVA pathway that showed reduced transcription are *MDPC1* (mevalonate diphosphate decarboxylase) and the further downstream *IPPI1* (isopentenyl diphosphate isomerase), the latter giving rise to two protein isoforms, one localized in the cytoplasm/peroxisomes and the other in plastids (Phillips et al. 2008). The plastidial MEP and carotenoid biosynthesis pathways are both transcriptionally downregulated in the *rgtb1* mutants (Supplementary Fig. S4), including genes for the regulatory enzymes deoxy-xylulose reductase and phytoene synthase (Banerjee and Sharkey 2014, Zhou et al. 2022, Bergman et al. 2024), and it is possible that the observed decrease in transcription of *IPPI1* may reflect downregulation of the chloroplast-localized isoform, not necessarily the cytoplasmic enzyme.

We also detected an increase in the transcription of *FPS2* (farnesyl diphosphate synthase), encoding the enzyme that performs the last common step in the biosynthesis of sterols and dolichols, but not of the *FPS1* gene (Closa et al. 2010, Keim et al. 2012, Figs 1 and 5), while transcription of the main GGPS gene, *GGPS11*, was decreased in *rgtb1* mutants when compared with WT plants. The interpretation of this result must take into account that the primers used in this study do not distinguish between the two translational isoforms encoded by this gene: the cytoplasmic GGPS11S and the plastidial GGPS11L (Ruiz-Sola et al. 2016a, 2016b). We did not study the transcription of the other putative cytoplasmic GGPS encoding genes, *GGPS3 (At2g18640)* or *GGPS4 (At2g23810)*, as their transcription is restricted to pollen (Kopcsayova and Vranova 2019; unibar.toronto). It is possible that the chloroplast isoform GGPS11L, which is responsible for the synthesis of photosynthetic pigments such as carotenoids and chlorophylls, is—at least in mature plants—present in much higher amounts than the cytoplasmic isoform GGPS11S (Ruiz-Sola et al. 2016a, 2016b). Similarly as for *IPPI1* above, the general decrease in *GGPS11* transcription could then result from a decrease in the production of the chloroplast isoform and have no connection to the situation in the cytoplasm.

At the same time, the transcription of genes encoding enzymes of the sterol pathway showed misregulation (schematic representation of the sterol biosynthetic pathway is presented on Fig. 8 and details on EC enzymatic activities in Table 2).

On the one hand, genes encoding the early sterol biosynthesis pathway (producing non-cyclic sterol precursors) were significantly downregulated: *SQE1, SQE2*, and *SQE3* (squalene epoxidases; Rasbery et al. 2007, Pose et al. 2009, Laranjeira et al. 2015). On the other hand, some genes encoding enzymes of the downstream sterol pathway were upregulated, in particular *CPI* (cyclopropyl isomerase, a plant-specific enzyme; Lovato et al. 2000, Rahier and Karst 2014), *SMT1* (sterol methyl transferase 1, which catalyzes the first methylation of the sterol side chain, also a plant-specific enzyme; Diener et al. 2000, Willemsen et al. 2003, Carland et al. 2010), *SMO1*–*3* (part of the sterol C-4 demethylating complex; Darnet and Rahier 2004, Song et al. 2019), and some late pathway genes, e.g. *SMT3*, *DWF1*, *CYP70A1,* and *CYP710A2* (Fig. 5, Choe et al. 1999, Carland et al. 2002, 2010, Morikawa et al. 2006, Arnqvist et al. 2008, Nakamoto et al. 2015, Youn et al. 2018). SMT3 is a low-expressed isoform of sterol methyltransferase, normally active only in a restricted number of organs (Carland et al. 2002). *DWF1* encodes a sterol C-24 reductase catalyzing the final conversion from 24-methylenecholesterol to campesterol (Choe et al. 1999, Youn et al. 2018). *CYP70A* forms a family of four genes in *Arabidopsis* and has a plant-specific activity of C-22 sterol desaturase (Morikawa et al. 2006, Arnqvist et al. 2008). These results, together with the data showing increased content and changed composition of sterols, point to an overall mis-regulation of sterol biosynthesis in *rgtb1* plants on the gene transcription level.

The transcription of genes encoding enzymes of the dolichol biosynthesis pathway was increased in the *rgtb1* mutants (Table 2, Fig. 5). This included the genes: *CPT3* (*cis*-prenyl transferase 3; Gawarecka et al. 2022), *PPRD2* (polyprenol reductase 2; Jozwiak et al. 2015; in tomato, this enzyme has been shown to be a key regulator of metabolite flow through the dolichol pathway; Van Gelder et al. 2021), and *DOK* (dolichol kinase, also known as *EVAN*; Kanehara et al. 2015, Lindner et al. 2015, Cho et al. 2017). These observations are consistent with the increase in dolichol content observed in the mutants (Fig. 4). Interestingly, we observed a reduction in the transcription of the *LEW1* gene encoding an accessory protein serving in dolichol precursor synthesis (Zhang et al. 2008).

### Cellular effects of sterol and dolichol pathway misregulation

The main regulatory enzyme of the MVA pathway, HMGR, but also many other sterol and dolichol biosynthesis enzymes, among them the ones we found transcriptionally affected, are intrinsic ER membrane proteins (Campos and Boronat 1995, Leivar et al. 2005, Ferrero et al. 2015, Jozwiak et al. 2015, Laranjeira et al. 2015, Lung et al. 2017). We hypothesized that the overall misregulation of cytoplasmic isoprenoid biosynthesis could derive from general ER dysfunction due to overaccumulation of vesicular cargo in case of Rab protein hypo-prenylation in the *rgtb1* mutants. Overall, changes in ER shape and functioning are for example obvious in yeast mutants lacking the RGTB activity (Newman and Ferro-Novick 1987). To test this hypothesis, we studied the functioning of the ER in *rgtb1* and WT plants at mature rosette stage.

**Figure 6:**
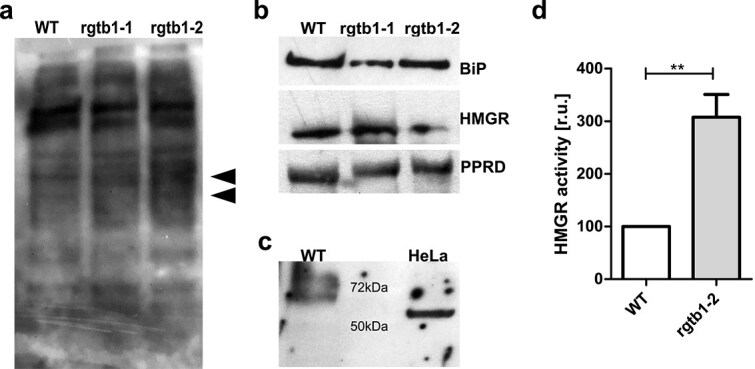
Analysis of endoplasmic reticulum functionality in *rgtb1* mutants. Total extracts from mature *rgtb1* and WT plants were prepared and analyzed by: (a) staining with concanavalin A recognizing mainly terminal α-mannose residues of glycosylated proteins and lipids, arrowheads point to the bands of changed intensity between samples. (b) immunodetection of ER proteins: BiP chaperone—a marker of ER stress, HMGR, the rate-limiting enzyme of the MVA pathway, PPRD, a key enzyme of dolichol biosynthesis. (c) anti-human HMGR antibody recognizes one band in *Arabidopsis* lysate of expected molecular mass of plant HMGR- 65 kDa. Equal amounts of protein were loaded in each lane. (d) HMGR activity in *rgtb1–2* mutant in comparison to WT plant extract. Enzymatic activity was measured as a difference in NADPH decay with/without HMG-CoA specific substrate in WT and *rgtb1–2* lysates normalized for protein amount. HMGR activity in *rgtb1–2* in each of three biological replicates is expressed as fraction of WT activity in the same experiment. Statistical analysis was performed using GraphPad PRISM 5.0 package by Student’s *t*-test against the H_0_ hypothesis that the values for WT and mutant are equal. Statistical significance: double asterisks denote *P* < 0.01.

We studied the amount of the ER BiP chaperone, an abundant stress-upregulated protein that takes part in the Unfolded Protein Response. We were unable to see any significant changes in the amount of this protein as estimated by immunostaining (Fig. 6b). Moreover, the glycosylation of proteins, as detected by Concanavalin A, a lectin that recognizes terminal α-mannose residues of glycosylated proteins and lipids, was not decreased or substantially changed in pattern in *rgtb1* in comparison to WT mature plants (Fig. 6a).

We then used Western blotting to check the amount of two key enzymes from the investigated pathways: HMGR for the MVA pathway and PPRD for dolichol formation (Fig. 6b). We detected no changes in the amounts of these enzymes in *rgtb1* plants in comparison to WT controls (Fig. 6b), despite the fact that the transcription of the genes *HMGR2* and *PPRD2* was elevated in the mutants (Fig. 5).

Finally, we also assayed the enzymatic activity of the HMGR protein in cell lysates from WT and *rgtb1–2* mutant plants. We used a spectrophotometric assay which measures the consumption of the reaction co-factor, NADPH, upon addition of the HMG-CoA substrate. The drawback of this assay is that the cell lysates contain multiple other active dehydrogenases which also consume NADPH, and the activity specific toward HMG-CoA is very low. Still, we observed a small but reproducible difference between samples with and without HMG-CoA that could be attributed to HMGR activity. This difference was more prominent in *rgtb1* lysates; the HMGR activity was increased approximately three times in the mutant plants (Fig. 6d).

**Figure 7:**
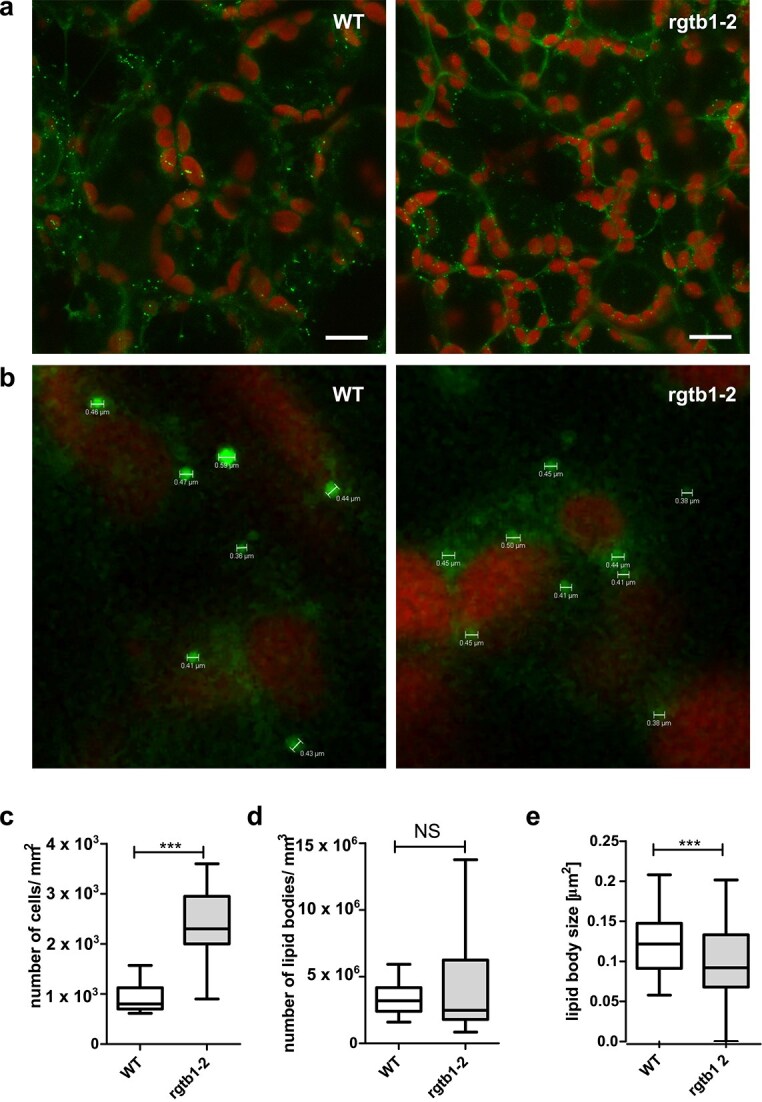
Analysis of lipid body number and morphology in WT and *rgtb1–2* leaf cells. Palisade mesophyll leaf cells from 6-week old plants grown in soil were vacuum-infiltrated with BODIPY 493/503 neutral lipid stain as described in section Materials and Methods. (a) Images of subsequent confocal layers were stacked. Green—BODIPY 493/503 stained lipid droplets, red—autofluorescence of chlorophyll in chloroplasts. Bar on both images corresponds to 1 *μ*m. (b) An example of measurements of lipid body diameters in WT and *rgtb1–2* mutant leaves. Microphotographs shown at the same scale. (c) Number of mesophyll cells per 1 mm^2^ of leaf in WT and *rgtb1–2* plants. (d) Number of lipid droplets per 1 mm^3^ of leaf mesophyll volume in WT and *rgtb1–2* plants. (e) Lipid droplet size in WT and *rgtb1–2* leaf mesophyll cells. Boxes correspond to mean ± SEM and whiskers show 95 to 5 percentile. Experiment was repeated for three independent plant cultures, at least three biological replicates each. All quantifications were taken from the same field 92.26 *μ*m × 92.26 *μ*m and z-stack from the same volume 92.26 *μ*m × 92.26 *μ*m × 7.43 *μ*m corresponding to 22 optical planes. Details of quantification are given in section Materials and Methods. Statistical analysis was performed using GraphPad PRISM 5.0 package by Student’s *t*-test against the H_0_ hypothesis that the values for WT and mutant are equal. Statistical significance: NS, non significant, triple asterisks denote *P* < 0.001.

Eukaryotic cells store excess sterols in the form of fatty acid esters in lipid droplets, together with triacylglycerols (Chapman et al. 2019, Cai and Horn 2025). In yeast, it has been documented that dolichols, probably in the form of esters, can be stored in lipid droplets (Currie et al. 2014, Hoffmann et al. 2017). We therefore decided to see if the excess amount of sterols and dolichols that we observed in *rgtb1* plants has an impact on lipid droplet number or size in the leaf cells of *rgtb1* mutants versus WT plants. To this end, we stained fragments of leaves of 6-week old soil grown plants with the neutral lipid stain BODIPY 493/503 and observed the localization of this marker in palisade mesophyll cells under the CLSM. The first striking observation was that the cells of the *rgtb1* mutant are significantly smaller than WT cells (so their density is approximately three times higher per mm^2^) (Fig. 7a and c). The size of the lipid droplets was lower but less uniform in *rgtb1* leaf mesophyll cells than in WT plants (Fig. 7b and e) but their number per volume of the tissue was similar for both studied genotypes (Fig. 7d). Taking into account that our biochemical studies determined an increased amount of isoprenoid lipids—sterols and dolichols—per amount of FW of tissue (Figs 2a and 4a), and that the water content and total lipid content of the tissue were not significantly different in WT than in *rgtb1* plants (Fig. 2e and f), we suppose that the lipid droplets in *rgtb1* mutants may have changes in lipid and/or protein composition when compared to WT plants.

## Discussion

Isoprenoid lipids in plants are synthesized on two biochemical routes: MVA, operating in the cytoplasm, and MEP, operating in chloroplasts, and each of these pathways produces important primary metabolites. Genes encoding enzymes of the MVA or MEP pathways are transcriptionally co-regulated to optimize the synthesis of the main products, i.e. sterols or photosynthetic pigments (Wille et al. 2004, Vranova et al. 2011). Each pathway independently undergoes strict internal feedback control to ensure proper regulation of metabolite flow through the appropriate branches leading to sterols, dolichols, and prenylated proteins in case of the MVA pathway, and mainly to photosynthetic pigments and hormones in case of the MEP pathway (for plants reviewed in Rodriguez-Concepcion and Boronat 2015, Pu et al. 2021, Bergman et al. 2024; for animals in Mitsche et al. 2015, Fracassi et al. 2019; and for biotechnologically important unicellular organisms in Li et al. 2021, Rinaldi et al. 2022, Dinday and Ghosh 2023). All cells and tissues need sterols, dolichols, and prenylated proteins for their basic metabolism, but depending on the environmental and nutritional conditions, as well as on developmental programs, they may differently equilibrate the activity of particular branches (Mitsche et al. 2015, Bergman et al. 2024). However, as studies on model membranes have shown, both sterols and dolichols, when present in excess, change the physical properties of membranes (Valtersson et al. 1985, Mongrand et al. 2010, Jarsch et al. 2014, Grosjean et al. 2015, Entova et al. 2019, Frallicciardi et al. 2022). Hence the increased amount of both sterols and dolichols, as well as changes in the proportions between particular compounds, might be detrimental to cells. For this reason, they are normally stored in an inert form, usually as acyl esters in lipid droplets in the cytoplasm (Ishinaga et al. 1992, Ferrer et al. 2017, Shimada et al. 2019, Guzha et al. 2023, Lopez-Tubau et al. 2025).

In this work, we show that *rgtb1* mutants, which have defects in protein geranylgeranylation, accumulate increased amounts of isoprenoid compounds: sterols, dolichols, triterpenoids. Since the mutant plants have smaller cells (as we observed for mesophyll palisade cells in leaves), we wondered if the higher surface-to-volume ratio could account for the increased lipid content, but the dry-weight-to-fresh-weight ratio as well as content of cell wall components and total lipids remain similar as in wild-type (WT) plants, confirming that the presented results reflect a true increase in the content of specific lipid categories.

In *rgtb1* plants, the total amount of 5-carbon isoprenoid moieties incorporated into sterols, dolichols, and non-sterol triterpenoids is significantly increased, suggesting that a larger pool of common precursors for these compounds could be available. This in turn would require an increase in the activity of the pathway’s rate-limiting enzyme, HMGR. This could potentially result from the increased transcription of *HMGR2* in *rgtb1* mutants, however, this result was not accompanied by an increased amount of the HMGR protein. Instead, we found an increase in HMGR enzymatic activity, which could account for an increased precursor pool. This result suggests that the regulation of protein activity could be dependent on proper protein prenylation. Furthermore, quantitatively, our results resemble the situation of *HMGR* overexpression in tobacco plants, which causes a 2–2.5-fold accumulation of total sterols (Harker et al. 2003, Holmberg et al. 2003)—a similar increase as in the *rgtb1* mutants—suggesting that this increase represents the overall capacity of the pathway under conditions of an increased precursor pool.

Why would an *rgtb1* mutation cause an increase in the substrates available for isoprenoid biosynthesis pathways? We consider two speculative explanations: that the *rgtb1* mutation could cause either an increase in the cytoplasmic pool of GGPP (a substrate of the RGT enzyme) or a decrease in the pool of a specific geranylgeranylated protein (geranylgeranylated Rab-GTPase, an RGT product), and that one of these compounds would be normally required for feedback control of HMGR activity which would then be defective in the mutant.

Regulation of HMGR activity by GGPP has been described for yeast and animal cells (Garza et al. 2009, Wangeline and Hampton 2018, Jun et al. 2025), in addition to the better described regulation by sterol pathway intermediate- and end-products (Song et al. 2005, Theesfeld and Hampton 2013, Faulkner et al. 2024). However, in plants, a direct involvement of GGPP in HMGR regulation has not been reported. It is also clear that GGPP levels in plant cells are the result of a complex net of interactions due to the involvement of plastids. Overall, GGPP turnover in plant cells is much higher than in animal cells, due to the high metabolic need for this compound in chloroplasts. The existence of two biosynthesis pathways for IPP/DMAPP and the fact that one GGPP synthase gene gives rise to two protein isoforms: one cytoplasmic and one chloroplast-localized (Ruiz-Sola et al. 2016a), complicate the situation even further.

As a result, even the actual origin of GGPP in the cytoplasm of plant cells is a matter of debate. In the *Nicotiana tabacum* BY-2 cell line, geranylgeraniol is derived solely from chloroplast synthesis and originates from the MEP pathway (Gerber et al. 2009, Chevalier et al. 2024). In *Arabidopsis*, the existence and functionality of GGPP synthase in the cytoplasm have been shown indisputably (Ruiz-Sola et al. 2016a). Moreover, feeding *Arabidopsis* plants with radioactive MVA results in its incorporation into cytoplasmic proteins (Gutkowska et al. 2004) and treatment of plants with the inhibitor mevinolin prevents protein prenylation (Swiezewska et al. 1993, Shipton et al. 1995), while treatment of whole plants of different species with the MEP pathway inhibitor clomazone did not interfere with the synthesis of geranylgeraniol (Weimer et al. 1992). Additionally, impairing cytoplasmic GGPP production by mutating *GGPS11* did not affect sterol levels (Ruiz-Sola et al. 2016a). Together, these results suggest that in our experimental model—mature, soil-grown *Arabidopsis* plants—the GGPP used for protein modification probably comes from an MVA-derived pool, but the involvement of GGPP imported from chloroplasts cannot be excluded.

For these reasons, we think that the negative signal for HMGR regulation would rather be a geranylgeranylated protein. The proteins RabD or RabC, which bind to ER and/or lipid droplet membranes, where the HMGR enzyme localizes, seem possible candidates (Zheng et al. 2005, Hashimoto et al. 2008, Pinheiro et al. 2009, Ge et al. 2022).

**Figure 8:**
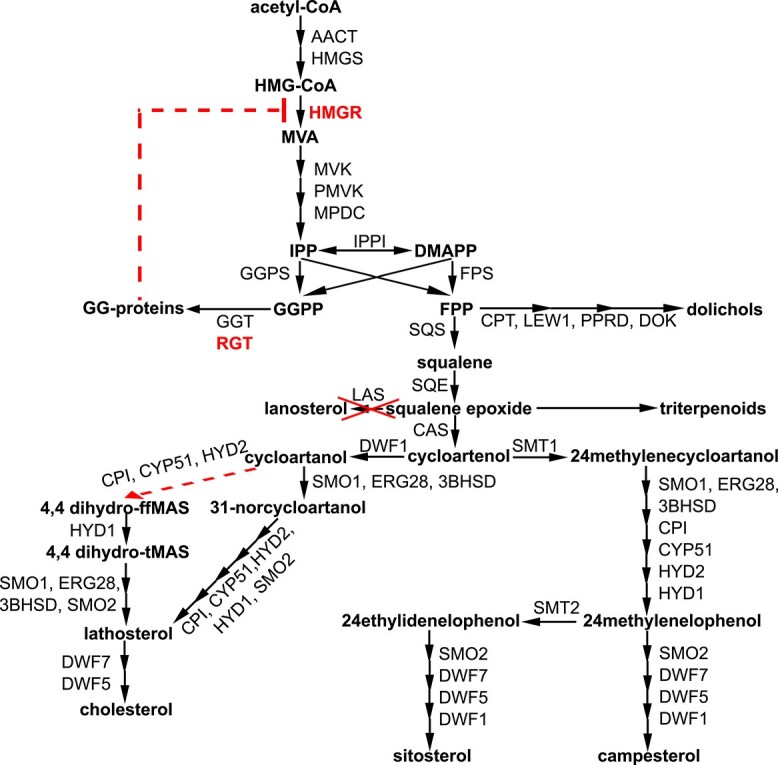
Summary of plant isoprenoid biosynthesis in *rgtb1* mutants. Graphical summary of the findings of the study. Each arrow represents a single enzymatic step. Well established pathways are marked in black. In red are shown the analyzed interventions (*rgtb1*, *las1*) and also red dashed lines denote putative new routes for biosynthesis or feedback inhibition. The names of the genes are shown in Table 2; the structural formulas of the main intermediates and products are shown in Fig. S1.

Based on the results presented in this article, we note a difference between the responses of the sterol and dolichol biosynthesis pathways to increased HMGR activity. Elevated levels of end products are detected for both pathways but the overall response is not the same: for the dolichol pathway, all assayed genes are upregulated (with the exception of only *LEW1*, coding for an accessory protein), while for the sterol pathway we observe misregulation, with some genes undergoing transcriptional induction and other repression. The misregulation is also visible in the accumulation of known intermediates as well as emergence of novel, unusual ones. This situation may be connected to differing modes of regulation of the pathways, one appropriate for the relatively simple, linear, dolichol pathway, and the other for the longer, more elaborate, highly branched system of sterol biosynthesis routes (Fig. 8).

The molar ratio of IPP in dolichol is 15 (or 16) isoprenoid units per molecule (compared to 6 units per sterol molecule), and in grams the amount of dolichols per tissue weight is very low (for WT *Arabidopsis* leaves ca. 0.5 *μ*g/g FW, compared to 300 *μ*g/g FW for total sterols), meaning that relatively there are very few dolichol molecules in the cell. However, in *rgtb1* mutants, the content of these isoprenoids increases five-fold, much more than the increase in sterols. Since dolichol synthesis is rather simple [based on the iterative elongation of the FPP molecule with IPP by a *cis*-prenyltransferase (Surmacz and Swiezewska 2011) followed by a reduction step catalyzed by PPRD (Jozwiak et al. 2015)], IPP overload in the cell might be directly mirrored by dolichol accumulation. PPRD2 was proposed to be the rate-limiting enzyme for this pathway in plants (Van Gelder et al. 2021), but in our study the slight induction of *PPRD2* seems to be sufficient to bear the substrate supply, as we did not observe an accumulation of polyprenol intermediates.

Also triterpenoids, derived from squalene epoxide (Fig. 8), are increased in the *rgtb1* mutants. Squalene epoxide can be cyclized to cycloartenol or lanosterol and as such is a key intermediate in sterol biosynthesis, but squalene can be oxidized also by other squalene-accepting enzymes (SQE-like; Laranjeira et al. 2015) and used by specific cyclases for the synthesis of secondary triterpenoid compounds such as α- and β-amyrin or lupeol (Segura et al. 2000, Phillips et al. 2006, Lodeiro et al. 2007, Ohyama et al. 2007, Shibuya et al. 2009, Xue et al. 2012), which were strongly elevated in our samples. These routes also seem to mirror directly the increase of upstream pathway activity, similarly to dolichol biosynthesis.

Surprisingly, we observed a reproducible decrease in the transcription of genes coding for functional squalene epoxidases: SQE1, SQE3, and the minor isoform SQE2 (Laranjeira et al. 2015). In earlier works, chemical inhibition of squalene epoxidase in tobacco cells, leading to a decrease in sterol biosynthesis, was found also to trigger a several-fold increase in HMGR enzyme activity (Hemmerlin and Bach 2000, Wentzinger et al. 2002), indicating feedback regulation exerted by pathway products and leading to the conclusion that squalene epoxidase is a second rate-limiting step in sterol biosynthesis. In our samples, the transcriptional downregulation of SQE genes neither cause accumulation of squalene nor did it impair sterol biosynthesis, showing that in the mutant cells, there is enough enzymatic activity left to sustain metabolite flow. Considering the postulated rate-limiting role of SQE in the pathway, it is possible that sterols provide a feedback signal for SQE transcription and that the transcriptional downregulation is itself a secondary effect of sterol accumulation.

The sterol biosynthesis pathway in *Arabidopsis* cells is a highly complex system with branches leading to several end products (various phytosterols, cholesterol, but also brassinosteroid hormones) and with the same enzymes catalyzing similar reactions in various branches using different substrates (Fig. 8). Introduction of the *rgtb1* mutation causes multiple perturbations in this complex system, leading to misregulation. Total sterol content is elevated and many genes are upregulated, but others are repressed, which together results in changes in the proportions between sterol products—in particular higher abundance of cholesterol—and accumulation of intermediates that are not detectable in WT plants. One example is dihydro-t-MAS, an intermediate found in animal cells on the route from lanosterol to cholesterol. Interestingly, despite the presence of this compound, we could not detect lanosterol in our samples, and we found that *A. thaliana* mutants deficient in the putative lanosterol synthase LAS1 show neither visible nor sterol-related phenotypes. These observations support the notion that lanosterol is not an indispensable metabolite in *Arabidopsis*, confirming data from (Sonawane et al. 2016) and contradicting data from (Suzuki et al. 2006, Ohyama et al. 2009). It is thus possible that dihydro-t-MAS could be synthesized from cycloartanol, through dihydro-ff-MAS, on a so far undescribed route (Fig. 8), and we further hypothesize that this might be the route that leads to cholesterol increase in *rgtb1* plants.

Another interesting observation is the significant increase in *DWF1* transcription in *rgtb1* plants. A broad ecological study has suggested an important role for DWF1 (sterol-Δ24-reductase) in crosstalk between various branches of sterol biosynthesis in plants (Zu et al. 2021). The human DWF1 homologue, DHCR24, shows activity toward a variety of sterol compounds: it converts the C-24 double bond in most cholesterol intermediates, including both early and late ones (Mitsche et al. 2015, Sharpe et al. 2020). This property of DHCR24 provides tissue-dependent flexibility to sterol biosynthesis in animals. Also in plants this type of DWF1-related tissue flexibility might exist, as suggested by the fact that cycloartenol, nor-cycloartenol and nor-cycloartanol are highly abundant sterols in *Arabidopsis* pollen (Villette et al. 2015). The route from cycloartenol to cholesterol through nor-cycloartanol is active in many plants (Ischebeck 2016, Furse et al. 2023). In this study, we observed accumulation of cycloartanol in the *rgtb1* samples, opening the possibility that under these conditions *Arabidopsis* DWF1 may be accepting cycloartenol as substrate, in a manner analogous to the reduction of nor-cycloartenol in pollen in WT plants.

Not only cycloartanol is accumulating in *rgtb1* plants, but also its precursor, cycloartenol, was detected in increased amounts. At the same time, 24-methylenecycloartanol—the product of cycloartenol methylation by SMT1, on the classic phytosterol pathway leading to campesterol and sitosterol—was detected in very low amounts, meaning that the increased transcription of *SMT1* is not sufficient to produce enough active enzyme for methylation of the accumulating cycloartenol. In earlier published experiments, an increase in HMGR activity (up to six times) led only to a moderate increase in end-product sterols in *Arabidopsis* but caused more than 100-fold accumulation of cycloartenol (Chappell et al. 1995). In our mutants, the upregulation of *DWF1* might be instead diverting this compound toward non-classical biosynthesis of cholesterol. It must be also kept in mind that sterol synthesis enzymes cooperate in large multi enzymatic complexes at ER membrane, which has been confirmed in plants for SMO complex (Mialoundama et al. 2013), but also other enzymes putatively interact, as has been described in non-plant eukaryotes (Mo et al. 2002, 2004a, 2004b, Mo and Bard 2005, Luu et al. 2015). If such interactions are taking place in plants, the accumulation of intermediates and side-products would depend on the enzymatic rate of the slowest enzyme in the complex.

Finally, it is also important to consider how the increased sterol and dolichol content influences *rgtb1* cells. It is likely that the excess lipids do not remain in ER membranes, where they are synthesized, because that would lead to ER stress (reflected by an accumulation of the BiP chaperone). A fraction of the accumulating sterols might reach the plasma membrane, but BODIPY 493/503 staining did not reveal any visible accumulation in this location, analogous to the sterol ester bodies detected previously in conditions of very high sterol accumulation in *Arabidopsis* cells (Shimada et al. 2019). Are the additional lipids packaged into lipid droplets (LD)? We found that the number of LDs per cell volume is unchanged in *rgtb1* mutants, but the average size is smaller. On one hand, this could be connected to the smaller size of *rgtb1* cells, but on the other hand, it could also mean that the additional sterols either cause tighter packing of LDs in *rgtb1* mutants or they change the biophysical properties of membranes and in this way influence growth and release of LDs from the ER. Indeed, the sterol-deficient *Arabidopsis* mutants *dwf5* and *dwf7* have been shown to contain larger LDs than WT cells (Yu et al. 2021), which is in line with this interpretation.

## Conclusion

In conclusion, we clearly observe changes in the functioning of cytoplasmic isoprenoid biosynthesis pathways in *A. thaliana* plants deficient in Rab-GTPase geranylgeranylation, manifesting in increased production of all assayed sterols (in particular, cholesterol) and dolichols as well as selected triterpenoids. Abnormal accumulation of some sterol pathway intermediates also occurs, showing that there is more crosstalk and flexibility in plant sterol synthesis than previously reported.

## Materials and Methods

### Plant material

Wild-type Col-0 plants were used as controls. *rgtb1–1* (SALK_015871) and *rgtb1–2* (SALK_125416) plants were used (Hala et al. 2010, Gutkowska et al. 2015) as mutants in RGT β subunit encoding gene *At5g12210*. T-DNA lines *las1–1* (SALK_128191C) and *las1–2* (SALK_058342) were used as mutants in lanosterol synthase gene *At3g450130*. All mutants were in Col-0 background and seeds were obtained from NASC. Plants were grown in soil, in the greenhouse, in standard long-day conditions (16 h light 23 °C/8 h darkness 16 °C). Mutant *rgtb1* plants were chosen based on the characteristic dwarf phenotype which co-segregated with the *rgtb1/rgtb1* genotype (Hala et al. 2010). *las1* mutants were chosen based on genotyping with primer pairs characteristic for the T-DNA insertion and the gene. Five-week old mature plants before bolting were harvested, rosettes with roots. 3–6 plants were used as biological replicates (more in case of *rgtb1*, because they are smaller). Material for all experiments came from six independent cultivations.

### Isolation of non-saponifiable lipids from plant material

Plant material (0.5–1 g) was homogenized in liquid nitrogen with mortar and pestle. Frozen powder was shaken in glass vials in 20 mL of a chloroform:methanol mixture (C/M; 1:1 v:v) for 24 h at room temperature in the dark and the extract was collected. This procedure was repeated three times. The material remaining in the vial after the three extractions was dried and weighed. This is referred to as the cell wall fraction. The obtained extracts from each sample were pooled, filtered into fresh glass vials, evaporated under a nitrogen stream at 50 °C and weighed on a precision analytical scale. This fraction is referred to as the total lipid fraction. This fraction was hydrolyzed in 5 mL of KOH in ethanol:toluene solution (0.384 g KOH, 0.384 mL water, 2.089 mL 96% ethanol, 2.527 mL toluene) for 1 h at boiling water bath. The non-saponifiable lipids were extracted three times with water:hexane mixture 1:1 (vol:vol), pooled fractions dried under the nitrogen stream and solubilized in 0.5 mL hexane. Further fractionation was performed on 1 mL silica-gel column equilibrated in hexane. To enable parallel analysis of compounds of varying polarity, we separated the lipid extracts on silica-gel columns and obtained fractions of increasing polarity: non-polar (elution with 2% ethyl ether in hexane), low-polar (10% ethyl ether in hexane), medium polar (20% ethyl ether in hexane), and polar (30% ethyl ether in hexane). All samples were dried under a nitrogen stream.

### HPLC–UV analysis of dolichols and squalene

Polyisoprenoid alcohols and squalene in fractions 10% or 2% ether ethyl in hexane, respectively were analyzed by high performance liquid chromatography with UV detector (HPLC-UV) on a 4.6 × 75 mm ZORBAX XDB-C18 (3.5 *μ*m) reversed-phase column (Agilent) using a Waters dual-pump apparatus, a Waters gradient programmer, and a Waters Photodiode Array Detector (spectrum range: 210–400 nm). The solvent composition and gradient for dolichol determination are described in Jozwiak et al. (2015). The chain length and identity of lipids were confirmed by comparison with external standards of a polyprenol mixture (Prenol-9–25) and dolichol mixture (Dolichol-17–21). Quantitative determination of polyisoprenoids was performed using the internal standards of Prenol 14 or Prenol 19 added in the amount of 10 *μ*g/sample at the initial lipid extraction stage. All standards came from Collection of Polyprenols, Institute of Biochemistry and Biophysics, Polish Academy of Sciences, (Warsaw, Poland). Squalene was analyzed on the same column and HPLC chromatograph in a gradient of solvent A (methanol:water (9:1, vol:vol) to solvent B (methanol:2-propanol:hexane (2:1:1, vol:vol:vol) as follows: 0% B to 50% B in 30 min, to 100% B in next 9 min, flow 1.5 mL/min. Quantitative determination of squalene was performed by comparison of a calibration curve made for 0, 2, 3.5, and 5 *μ*g of squalene standard (Sigma). Integration of the HPLC–UV chromatograms was performed with the Empower software (Waters).

### GC-FID and GC–MS analysis of sterols and their biosynthetic precursors

Phytosterols are relatively polar compounds and were mostly found in the fractions eluted with 20% and 30% ethyl ether in hexane, which were further studied by gas chromatography with flame ionization detector (GC-FID). Sitosterol, campesterol, and cholesterol were the most abundant sterols in samples from WT plants and were present in both 30% ethyl ether elution and 20% ethyl ether elution, we calculated the total amount of these compounds as a sum from these two fractions. The sterol-containing fractions of 30% ethyl ether in hexane and less polar sterol precursors of 20% of ethyl ether in hexane were analyzed at Warsaw University, Faculty of Biology (Warsaw, Poland) using gas chromatography with mass spectrometry detector (GC–MS) (7890 A Agilent gas chromatograph with an FID, equipped with a 5975 C VL MSD—Perlan) as described in (Hoffman-Sommer et al. 2025). Samples dissolved in a mixture of diethyl ether/methanol (1:1, vol/vol) were applied in a volume of 3 *μ*L using a 1:3 split injection on the Ultra-Inert GC column HP-5MS UI (30 m × 0.25 mm i.d.; film thickness 0.25 *μ*m—Agilent Technologies, Santa Clara, CA, USA). The carrier gas was helium at a flow rate of 1 mL/min. The following program was carried out: 160 °C for 2 min, then an increase of 5° per minute up to 280 °C, which were held for 24 min. The following settings were used: inlet and FID temperature: 290 °C, MS transfer line temperature: 275 °C, quadrupole temperature: 150 °C, ion source temperature: 230 °C, EI: 70 eV, *m/z* range: 33–500, FID gases: H_2_ from a hydrogen generator at 30 mL/min and air at 400 mL/min. Alternatively the analysis was made at the Institute of Organic Chemistry, PAS (Warsaw, Poland) on Agilent 7890A gas chromatograph coupled with Agilent 5975C single quadrupole mass spectrometer with EI ion source (Agilent Technologies, Santa Clara, CA, USA) equipped with a 30 m long HP-5 MS column, with 0.25 mm inner diameter, and 0.25 *μ*m stationary phase film thickness as described in (Warchol et al. 2016). One microliter of lipid sample (hexane extract) was injected and the column temperature was set at 150 °C for 5 min, next it was increased to 300 °C with the ramp of 5 °C/min and the final temperature was set at 300 °C for 30 min. Helium was used as a carrier gas and the flow rate was set at 1 mL/min. The scan mode of 33–600 *m/z* was used to monitor mass spectra. Sterols were identified by comparing their spectra with the databases (Wiley 9th ED. and NIST 2008 Lib. SW Version 2010) or on comparisons of the obtained retention times and mass spectra with those for external standards. FID chromatograms were integrated using the ChemStation E.02.012010 software. Area under the signals of sterol and of internal control (cholestanol) served to calculate the amount of sterols. All internal and external standards of analytical grade were purchased from Sigma Aldrich or AvantiPolar Lipids.

### UPLC-MS analysis of sterol precursors

Instrumentation consisted of Waters Acquity Ultra Performance Liquid Chromatograph coupled with Waters TQ-S triple-quadrupole mass spectrometer. For the instrument control and data acquisition, MassLynx software was used. UPLC–MS–MS analysis were performed in positive atmospheric pressure chemical ionization mode. Mass spectrometer operated in MRM. For chromatographic separation we applied UPLC Phenyl-Hexyl column (100 × 2.1 mm, 1.7 *μ*m, Waters) thermostatted at 70 °*C. *Mobile**phase A was Mili-Q water with addition of 1 mL of 0.01% formic acid in water, and mobile phase B was 0.01% formic acid in methanol. The flow rate of mobile phase was set at 0.45 mL/min and the injection volume was 3 *μ*L. The gradient scheme was: 25% B initially, increase to 100% B at 4 min. At 5 min, the mobile phase reverted to initial condition (70% B). The total analysis time was 6 min including re-equilibration time. For all analyzed compounds, mass spectrometer optimized settings were as follows: capillary voltage = 3.0 kV, desolvation temperature = 500 °C, desolvation gas flow = 900 L/h, cone gas flow = 150 L/h, nebulizer gas pressure = 7.0 bar, source temperature = 150 °C. All standards of analytical grade were purchased from Sigma Aldrich or AvantiPolar Lipids.

### RT–qPCR analysis

Five-week-old plants grown in soil were harvested, roots washed extensively in water, and snap-frozen in liquid nitrogen. Homogenization was performed in liquid nitrogen by mortar and pestle. About 65 mg of powdered tissue was weighted. RNA from plant samples was prepared with GeneJet RNA Purification Kit (Thermo Fisher Scientific) according to manufacturer’s instructions for the plant material. Equilibrated amount of RNA was treated with TurboDNaze free Kit (Thermo Fisher Scientific) to remove genomic DNA and finally cDNA was prepared with RevertAid kit (Thermo Fisher Scientific) at 42 °C for 1 h with oligo dT primer using 1 *μ*g of RNA per reaction. RT-qPCR reactions were performed in 384 well-plates (Roche) in LightCycler 480 (Roche) equipped with LightCycler 480S.W 1.5 package in technical duplicates for each cDNA sample. For RT-qPCR analysis, reaction mixtures contained 2.9 *μ*L miliQ water, 5 *μ*L SYBR green (Roche), 0.05 *μ*L of 100 *μ*M forward primer, and 0.05 *μ*L of 100 *μ*M reverse primer and 2 *μ*L of 10-fold diluted cDNA. For each cDNA sample a referential gene *PP2AA3* was used as a normalization standard. Primer sequences are listed in Supplementary Table S2. Primers were designed in Primer-BLAST (www.ncbi.nlm.nih.gov) to amplify the exon-exon junction region of 100–150 bp length, giving only one specific product located close to the 3′ end of the analyzed gene. Mean Cp values were normalized to the expression of *PP2A*. Experiment was performed for at least three biological replicates.

### Immunological analysis of selected protein amount

Five-week-old plants grown in soil were harvested, roots washed extensively in water, and snap-frozen in liquid nitrogen. Homogenization was performed in liquid nitrogen by mortar and pestle. About 100 mg of powdered tissue was weighed and suspended in 0.5 mL of homogenization buffer containing: 50 mM Tris pH 8.0, 100 mM NaCl, 1 mM EDTA, 1 mM DTT, 20% glycerol, and 0.5% SDS. Homogenates were centrifuged 30 min at 14 000 rpm at 4 °C. The supernatant was normalized for protein content by the Bradford method (ThermoFisher). Equal amount of protein was loaded to each lane of 12% SDS-PAGE gel. The gels were blotted on Immobilon-P polyvinylidene fluoride (PVDF) transfer membrane (for lectin staining) or ProTrans Nitrocellulose transfer membrane (for protein immunodetection). The PVDF membranes were blocked in RIPA buffer and nitrocellulose membranes in 5% non-fat milk in PBS overnight at 4 °C. The antibodies used were as follows: anti-*Arabidopsis* BiP AS09 481 rabbit polyclonal antibody (Agrisera) at 1:2000 solution, anti-human HMGR 07–457 rabbit polyclonal antibody (Upstate) at 1:1000 solution (a kind gift of prof. Valentina Pallotini, Universita di Sapienza, Rome, Italy), this antibody recognizes one band in *Arabidopsis* lysate at expected molecular mass- about 65 kDa and one in human HeLa cell lysate- at the expected mass of HMGR cytoplasmic domain of 55 kDa. anti-human HMGR ab242315 mouse monoclonal antibody (Abcam) at 1:1000 dilution (a kind gift of prof. Marco Segatto, University of Molise, Italy) did not react with plant protein. Anti-*Arabidopsis* PPRD rabbit polyclonal antibody (custom antibody raised against the peptide 202–216 of PPRD2: C + FIANGKSHTSAPEFN, Kaneka Eurogentec SA, Belgium, described by Piłka et al., *manuscript in preparation*) at 1:2000 dilution recognized in plant lysate only a band of expected molecular mass of about 40 kDa. The goat anti-rabbit-IgG-HRP secondary antibody (Sigma, A0545) was used at a concentration of 1:4000. Staining for protein glycosylation was performed with lectin conjugated to HRP: concanavalin A (ThermoFisher) in concentration 1:1000 for 1 h. The signal was developed on a photographic membrane (Hyperfilm, Amersham) with the use of chemiluminescence kit SuperSignal Plus WestPico (Invitrogen).

### HMGR activity assay

Five-week old plants grown in soil (rosettes and roots) were homogenized with mortar and pestle in liquid nitrogen with 200 *μ*L buffer 50 mM Tris 7.3, 50 mM KCl, 0.2% Triton X-100, 10 mM DTT and protease inhibitor cocktail (Plant mini, EDTA free, Roche) as described in Campos et al. 2014. Each sample included material from at least three different plants (in case of mutants more) and weighed about 100 *μ*g. Homogenates were centrifuged at 2000 *g* at 4 °C for 10 min to discard cell debris (according to Campos et al. 2014, in mature leaves the HMGR protein remains in the pellet after centrifugation at 16 000 *g*). HMGR activity determination was carried out in 100 *μ*L buffer containing 50 *μ*L of extract, 5 mM EDTA, 2.5 mM NADPH, and 0.3 mM HMG-CoA. In control samples, the specific substrate HMG-CoA was omitted. Reactions were carried out at 30 °C with shaking in 96-well U-shaped-bottom clear microplates (Greiner) in technical triplicate. The decrease in NADPH fluorescence at 340 nm was recorded every 1 min for 1 h in a VarioscanLux microplate reader (ThermoFisher Scientific). The activity per 1 min was normalized for the protein amount in the sample, determined by Bradford method. Activity of non-related NADPH-dependent dehydrogenases (control without HMG-CoA substrate) was subtracted from the measured value. The experiment was carried out for three independent biological replicates. The value for samples from WT plants was treated as reference for the values obtained for the *rgtb1* mutants from the same biological replicate.

### Confocal laser scanning microscopy

Fragments of leaves from mature, 6-week-old soil-grown plants of either the WT or *rgtb1–2* genotype were cut with a lancet and incubated in 10 mM PIPES buffer (pH adjusted to 6.8 using KOH) containing 3.8 *μ*M BODIPY 493/503 (Sigma Aldrich, 790 389), added just before use. Samples were incubated for 15 min in the dark at room temperature then washed with fresh buffer and mounted on slides. Imaging was performed no earlier than 30 min and no later than 1 h after mounting.

All imaging was conducted using a Leica Stellaris 8 FALCON confocal microscope equipped with an 80 MHz pulsed white light laser tunable in 1 nm increments from 440 to 790 nm. The laser was set to 1% of 85% of the maximum power at 486 nm for the excitation of BODIPY 493/503 and chlorophyll. The laser was turned on 1 h prior to data acquisition to allow for its stabilization. Note that laser intensity was relative so the exact laser power at the sample was not determined.

Emission was detected using HyD S detectors with spectral ranges set to 491–550 nm (gain 50.0) for BODIPY 493/503 and 630–752 nm (gain 2.5) for chlorophyll, and was displayed in false green and red colors, respectively. Imaging was performed using an HC PL APO CS2 63×/1.2 NA water immersion objective with a digital zoom of 2.0, resolution of 1128 × 1128 pixels, and a pixel dwell time of 0.9 *μ*s. Line averaging (×2) was used for z-series collection, with pixel sizes of 0.08 *μ*m in the xy-plane and 0.35 *μ*m in the z-dimension. The pinhole size was set to 1 Airy unit and imaging was optimized according to the Nyquist criterion in LAS X software (version 4.8.0.28989) for a 580 nm wavelength. All acquisition settings were kept constant across all collected datasets. The surface of scanning had dimensions 92.26 *μ*m × 92.26 *μ*m and the depth of scanning was equal for all samples 7.43 *μ*m corresponding to 22 optical planes.

For the measurement of lipid droplet diameters (Fig. 7b), images were analyzed in LAS X software. Statistical analyses of cell numbers, lipid bodies number, and sizes (Fig. 7c–e) were performed using FIJI/ImageJ (ver. 2.14.0/1.54f; https://imagej.net/software/fiji/). Data were obtained from single scans of z-stacks, pre-processed using a Median filter (radius = 1.0) and Yen thresholding. Graphs were generated in GraphPad PRISM (version 5.0). Z-stack projections were stitched using FigureJ plugin (https://imagej.net/plugins/figurej) (Fig. 7a). Microscopy was performed in the Laboratory of Fluorescence Microscopy in IBB PAS, Poland.

## Supplementary Material

SuppFig1_Chemical_formulas_pcaf166

SuppFig2_LC-MS-MRS_pcaf166

SuppFig3_las1_mutant_pcaf166

SuppFig4_MEP_carotenoid_expression_pcaf166

Table_S1_names_of_genes_MEP_and_carotenoid_pcaf166

TableS2_primers_pcaf166

Supplementary_materials_pcaf166

## Data Availability

The data underlying this article will be shared on reasonable request to the corresponding author.

## References

[ref1] Arnqvist, L., Persson, M., Jonsson, L., Dutta, P.C. and Sitbon, F. (2008) Overexpression of CYP710A1 and CYP710A4 in transgenic Arabidopsis plants increases the level of stigmasterol at the expense of sitosterol. Planta 227: 309–317. 17909855 10.1007/s00425-007-0618-8

[ref2] Banerjee, A. and Sharkey, T.D. (2014) Methylerythritol 4-phosphate (MEP) pathway metabolic regulation. Nat. Prod. Rep. 31: 1043–1055. 24921065 10.1039/c3np70124g

[ref3] Bergman, M.E., Kortbeek, R.W.J., Gutensohn, M. and Dudareva, N. (2024) Plant terpenoid biosynthetic network and its multiple layers of regulation. Prog. Lipid Res. 95: 101287. 38906423 10.1016/j.plipres.2024.101287

[ref4] Buschhaus, C. and Jetter, R. (2012) Composition and physiological function of the wax layers coating Arabidopsis leaves: beta-amyrin negatively affects the intracuticular water barrier. Plant Physiol. 160: 1120–1129. 22885935 10.1104/pp.112.198473 PMC3461534

[ref5] Cai, Y. and Horn, P.J. (2025) Packaging “vegetable oils”: insights into plant lipid droplet proteins. Plant Physiol. 197: kiae533. 39566075 10.1093/plphys/kiae533

[ref6] Campos, N. and Boronat, A. (1995) Targeting and topology in the membrane of plant 3-hydroxy-3-methylglutaryl coenzyme a reductase. Plant Cell 7: 2163–2174. 8718626 10.1105/tpc.7.12.2163 PMC161070

[ref7] Campos, N., Arró, M., Ferrer, A. and Boronat, A. (2014) Determination of 3-Hydroxy-3-methylglutaryl CoA reductase activity in plants. Plant Isoprenoids: Methods Protoc. Methods Mol. Biol. 1153: 21–40. 10.1007/978-1-4939-0606-2_3 24777788

[ref8] Carland, F.M., Fujioka, S., Takatsuto, S., Yoshida, S. and Nelson, T. (2002) The identification of CVP1 reveals a role for sterols in vascular patterning. Plant Cell 14: 2045–2058. 12215504 10.1105/tpc.003939 PMC150754

[ref9] Carland, F., Fujioka, S. and Nelson, T. (2010) The sterol methyltransferases SMT1, SMT2, and SMT3 influence Arabidopsis development through nonbrassinosteroid products. Plant Physiol. 153: 741–756. 20421456 10.1104/pp.109.152587 PMC2879779

[ref10] Chapman, K.D., Aziz, M., Dyer, J.M. and Mullen, R.T. (2019) Mechanisms of lipid droplet biogenesis. Biochem. J. 476: 1929–1942. 31289128 10.1042/BCJ20180021

[ref11] Chappell, J., Wolf, F., Proulx, J., Cuellar, R. and Saunders, C. (1995) Is the reaction catalyzed by 3-Hydroxy-3-Methylglutaryl coenzyme a reductase a rate-limiting step for isoprenoid biosynthesis in plants? Plant Physiol. 109: 1337–1343. 12228673 10.1104/pp.109.4.1337 PMC157667

[ref12] Chevalier, Q., Huchelmann, A., Debie, P., Mercier, P., Hartmann, M., Vonthron-Senecheau, C., et al. (2024) Methyl-Jasmonate functions as a molecular switch promoting cross-talk between pathways for the biosynthesis of isoprenoid backbones used to modify proteins in plants. Plants (Basel) 13: 1110. 38674519 10.3390/plants13081110 PMC11055089

[ref13] Cho, Y., Yu, C.Y., Nakamura, Y. and Kanehara, K. (2017) Arabidopsis dolichol kinase AtDOK1 is involved in flowering time control. J. Exp. Bot. 68: 3243–3252. 28379398 10.1093/jxb/erx095 PMC5853391

[ref14] Cho, S.H., Toth, K., Kim, D., Vo, P.H., Lin, C.H., Handakumbura, P.P., et al. (2022) Activation of the plant mevalonate pathway by extracellular ATP. Nat. Commun. 13: 450. 35064110 10.1038/s41467-022-28150-w PMC8783019

[ref15] Choe, S., Dilkes, B.P., Gregory, B.D., et al. (1999) The Arabidopsis dwarf1 mutant is defective in the conversion of 24-methylenecholesterol to campesterol in brassinosteroid biosynthesis. Plant Physiol. 119: 897–907. 10069828 10.1104/pp.119.3.897 PMC32104

[ref16] Closa, M., Vranova, E., Bortolotti, C., Bigler, L., Arro, M., Ferrer, A., et al. (2010) The *Arabidopsis thaliana* FPP synthase isozymes have overlapping and specific functions in isoprenoid biosynthesis, and complete loss of FPP synthase activity causes early developmental arrest. Plant J. 63: 512–525. 20497375 10.1111/j.1365-313X.2010.04253.x

[ref17] Currie, E., Guo, X., Christiano, R., Chitraju, C., Kory, N., Harrison, K., et al. (2014) High confidence proteomic analysis of yeast LDs identifies additional droplet proteins and reveals connections to dolichol synthesis and sterol acetylation. J. Lipid Res. 55: 1465–1477. 24868093 10.1194/jlr.M050229 PMC4076087

[ref18] Darnet, S. and Rahier, A. (2004) Plant sterol biosynthesis: identification of two distinct families of sterol 4alpha-methyl oxidases. Biochem. J. 378: 889–898. 14653780 10.1042/BJ20031572 PMC1224014

[ref19] Darnet, S., Martin, L.B.B., Mercier, P., Bracher, F., Geoffroy, P. and Schaller, H. (2020) Inhibition of phytosterol biosynthesis by azasterols. Molecules 25: 1111. 32131509 10.3390/molecules25051111 PMC7179204

[ref20] Diener, A.C., Li, H., Zhou, W., Whoriskey, W.J., Nes, W.D. and Fink, G.R. (2000) Sterol methyltransferase 1 controls the level of cholesterol in plants. Plant Cell 12: 853–870. 10852933 10.1105/tpc.12.6.853 PMC149089

[ref21] Dinday, S. and Ghosh, S. (2023) Recent advances in triterpenoid pathway elucidation and engineering. Biotechnol. Adv. 68: 108214. 37478981 10.1016/j.biotechadv.2023.108214

[ref22] Elsabrouty, R., Jo, Y., Hwang, S., Jun, D.J. and DeBose-Boyd, R.A. (2021) Type 1 polyisoprenoid diphosphate phosphatase modulates geranylgeranyl-mediated control of HMG CoA reductase and UBIAD1. Elife 10: e64688. 34842525 10.7554/eLife.64688 PMC8641950

[ref23] Enjuto, M., Balcells, L., Campos, N., Caelles, C., Arro, M. and Boronat, A. (1994) Arabidopsis thaliana contains two differentially expressed 3-hydroxy-3-methylglutaryl-CoA reductase genes, which encode microsomal forms of the enzyme. Proc. Natl. Acad. Sci. USA 91: 927–931. 8302869 10.1073/pnas.91.3.927 PMC521426

[ref24] Entova, S., Guan, Z. and Imperiali, B. (2019) Investigation of the conserved reentrant membrane helix in the monotopic phosphoglycosyl transferase superfamily supports key molecular interactions with polyprenol phosphate substrates. Arch. Biochem. Biophys. 675: 108111. 31563509 10.1016/j.abb.2019.108111 PMC6909930

[ref25] Erffelinck, M.L. and Goossens, A. (2018) Review: endoplasmic reticulum-associated degradation (ERAD)-dependent control of (tri)terpenoid metabolism in plants. Planta Med. 84: 874–880. 29906815 10.1055/a-0635-8369

[ref26] Faulkner, R. and Jo, Y. (2022) Synthesis, function, and regulation of sterol and nonsterol isoprenoids. Front. Mol. Biosci. 9: 1006822. 36275615 10.3389/fmolb.2022.1006822 PMC9579336

[ref27] Faulkner, R.A., Yang, Y., Tsien, J., Qin, T. and DeBose-Boyd, R.A. (2024) Direct binding to sterols accelerates endoplasmic reticulum-associated degradation of HMG CoA reductase. Proc. Natl. Acad. Sci. USA 121: e2318822121. 38319967 10.1073/pnas.2318822121 PMC10873557

[ref28] Ferrer, A., Altabella, T., Arro, M. and Boronat, A. (2017) Emerging roles for conjugated sterols in plants. Prog. Lipid Res. 67: 27–37. 28666916 10.1016/j.plipres.2017.06.002

[ref29] Ferrero, S., Grados-Torrez, R.E., Leivar, P., Antolin-Llovera, M., Lopez-Iglesias, C., Cortadellas, N., et al. (2015) Proliferation and morphogenesis of the endoplasmic reticulum driven by the membrane domain of 3-hydroxy-3-methylglutaryl coenzyme a reductase in plant cells. Plant Physiol. 168: 899–914. 26015445 10.1104/pp.15.00597 PMC4741317

[ref31] Fracassi, A., Marangoni, M., Rosso, P., Pallottini, V., Fioramonti, M., Siteni, S., et al. (2019) Statins and the brain: more than lipid lowering agents? Curr. Neuropharmacol. 17: 59–83. 28676012 10.2174/1570159X15666170703101816 PMC6341496

[ref32] Frallicciardi, J., Melcr, J., Siginou, P., Marrink, S.J. and Poolman, B. (2022) Membrane thickness, lipid phase and sterol type are determining factors in the permeability of membranes to small solutes. Nat. Commun. 13: 1605. 35338137 10.1038/s41467-022-29272-x PMC8956743

[ref33] Furse, S., Martel, C., Yusuf, A., Shearman, G.C., Koch, H. and Stevenson, P.C. (2023) Sterol composition in plants is specific to pollen, leaf, pollination and pollinator. Phytochemistry 214: 113800. 37532086 10.1016/j.phytochem.2023.113800 PMC10493607

[ref34] Garza, R.M., Tran, P.N. and Hampton, R.Y. (2009) Geranylgeranyl pyrophosphate is a potent regulator of HRD-dependent 3-hydroxy-3-methylglutaryl-CoA reductase degradation in yeast. J. Biol. Chem. 284: 35368–35380. 19776008 10.1074/jbc.M109.023994 PMC2790966

[ref35] Gawarecka, K., Siwinska, J., Poznanski, J., et al. (2022) cis-prenyltransferase 3 and alpha/beta-hydrolase are new determinants of dolichol accumulation in *Arabidopsis*. Plant Cell Environ. 45: 479–495. 34778961 10.1111/pce.14223 PMC9300173

[ref36] Ge, S., Zhang, R.X., Wang, Y.F., Sun, P., Chu, J., Li, J., et al. (2022) The *Arabidopsis* Rab protein RABC1 affects stomatal development by regulating lipid droplet dynamics. Plant Cell 34: 4274–4292. 35929087 10.1093/plcell/koac239 PMC9614440

[ref37] Gerber, E., Hemmerlin, A., Hartmann, M., et al. (2009) The plastidial 2-C-methyl-D-erythritol 4-phosphate pathway provides the isoprenyl moiety for protein geranylgeranylation in tobacco BY-2 cells. Plant Cell 21: 285–300. 19136647 10.1105/tpc.108.063248 PMC2648074

[ref38] Grosjean, K., Mongrand, S., Beney, L., Simon-Plas, F. and Gerbeau-Pissot, P. (2015) Differential effect of plant lipids on membrane organization: specificities of phytosphingolipids and phytosterols. J. Biol. Chem. 290: 5810–5825. 25575593 10.1074/jbc.M114.598805 PMC4342490

[ref39] Gutkowska, M., Bienkowski, T., Hung, V.S., Wanke, M., Hertel, J., Danikiewicz, W., et al. (2004) Proteins are polyisoprenylated in Arabidopsis thaliana. Biochem. Biophys. Res. Commun. 322: 998–1004. 15336563 10.1016/j.bbrc.2004.08.025

[ref40] Gutkowska, M., Wnuk, M., Nowakowska, J., Lichocka, M., Stronkowski, M.M. and Swiezewska, E. (2015) Rab geranylgeranyl transferase beta subunit is essential for male fertility and tip growth in Arabidopsis. J. Exp. Bot. 66: 213–224. 25316062 10.1093/jxb/eru412 PMC4265159

[ref41] Gutkowska, M., Kaus-Drobek, M., Hoffman-Sommer, M., et al. (2021) Impact of C-terminal truncations in the *Arabidopsis* Rab escort protein (REP) on REP-Rab interaction and plant fertility. Plant J. 108: 1400–1421. 34592024 10.1111/tpj.15519 PMC9293207

[ref42] Guzha, A., Whitehead, P., Ischebeck, T. and Chapman, K.D. (2023) Lipid droplets: packing hydrophobic molecules within the aqueous cytoplasm. Annu. Rev. Plant Biol. 74: 195–223. 36413579 10.1146/annurev-arplant-070122-021752

[ref43] Hala, M. and Zarsky, V. (2019) Protein prenylation in plant stress responses. Molecules 24: 3906. 31671559 10.3390/molecules24213906 PMC6866125

[ref44] Hala, M., Soukupova, H., Synek, L. and Zarsky, V. (2010) *Arabidopsis* RAB geranylgeranyl transferase beta-subunit mutant is constitutively photomorphogenic, and has shoot growth and gravitropic defects. Plant J. 62: 615–627. 20180921 10.1111/j.1365-313X.2010.04172.x

[ref45] Harker, M., Holmberg, N., Clayton, J.C., Gibbard, C.L., Wallace, A.D., Rawlins, S., et al. (2003) Enhancement of seed phytosterol levels by expression of an N-terminal truncated *Hevea brasiliensis* (rubber tree) 3-hydroxy-3-methylglutaryl-CoA reductase. Plant Biotechnol. J. 1: 113–121. 17147748 10.1046/j.1467-7652.2003.00011.x

[ref46] Hashimoto, K., Igarashi, H., Mano, S., Takenaka, C., Shiina, T., Yamaguchi, M., et al. (2008) An isoform of *Arabidopsis* myosin XI interacts with small GTPases in its C-terminal tail region. J. Exp. Bot. 59: 3523–3531. 18703495 10.1093/jxb/ern202 PMC2561144

[ref47] Hemmerlin, A. and Bach, T.J. (2000) Farnesol-induced cell death and stimulation of 3-hydroxy-3-methylglutaryl-coenzyme a reductase activity in tobacco cv bright yellow-2 cells. Plant Physiol. 123: 1257–1268. 10938345 10.1104/pp.123.4.1257 PMC59085

[ref48] Hemmerlin, A., Harwood, J.L. and Bach, T.J. (2012) A raison d'etre for two distinct pathways in the early steps of plant isoprenoid biosynthesis? Prog. Lipid Res. 51: 95–148. 22197147 10.1016/j.plipres.2011.12.001

[ref49] Hoffmann, R., Grabinska, K., Guan, Z., Sessa, W.C. and Neiman, A.M. (2017) Long-chain polyprenols promote spore wall formation in *Saccharomyces cerevisiae*. Genetics 207: 1371–1386. 28978675 10.1534/genetics.117.300322 PMC5714454

[ref50] Hoffman-Sommer, M., Pilka, N., Anielska-Mazur, A., Nowakowska, J., Kozieradzka-Kiszkurno, M., Paczkowski, C., et al. (2025) The TRAPPC8/TRS85 subunit of the *Arabidopsis* TRAPPIII tethering complex regulates endoplasmic reticulum function and autophagy. Plant Physiol. 197: kiaf042. 40084709 10.1093/plphys/kiaf042 PMC11907232

[ref51] Holmberg, N., Harker, M., Wallace, A.D., Clayton, J.C., Gibbard, C.L. and Safford, R. (2003) Co-expression of N-terminal truncated 3-hydroxy-3-methylglutaryl CoA reductase and C24-sterol methyltransferase type 1 in transgenic tobacco enhances carbon flux towards end-product sterols. Plant J. 36: 12–20. 12974807 10.1046/j.1365-313x.2003.01851.x

[ref52] Husselstein-Muller, T., Schaller, H. and Benveniste, P. (2001) Molecular cloning and expression in yeast of 2,3-oxidosqualene-triterpenoid cyclases from *Arabidopsis thaliana*. Plant Mol. Biol. 45: 75–92. 11247608 10.1023/a:1006476123930

[ref53] Ischebeck, T. (2016) Lipids in pollen - they are different. Biochim. Biophys. Acta 1861: 1315–1328. 27033152 10.1016/j.bbalip.2016.03.023

[ref54] Ishinaga, M., Yamauchi, T., Egusa, K. and Kuroda, K. (1992) Changes of dolichol and dolichyl fatty acyl esters during the germination and development of soybeans. Biochem. Cell Biol. 70: 466–469. 1449711 10.1139/o92-071

[ref55] Jarsch, I.K., Konrad, S.S., Stratil, T.F., Urbanus, S.L., Szymanski, W., Braun, P., et al. (2014) Plasma membranes are subcompartmentalized into a plethora of coexisting and diverse microdomains in *Arabidopsis* and *Nicotiana benthamiana*. Plant Cell 26: 1698–1711. 24714763 10.1105/tpc.114.124446 PMC4036580

[ref56] Jozwiak, A., Gutkowska, M., Gawarecka, K., Surmacz, L., Buczkowska, A., Lichocka, M., et al. (2015) Polyprenol reductase2 deficiency is lethal in *Arabidopsis* due to male sterility. Plant Cell 27: 3336–3353. 26628744 10.1105/tpc.15.00463 PMC4707453

[ref57] Jun, D.J., Schumacher, M.M., Jo, Y., Faulkner, R.A., Yang, Y., Tsien, J., et al. (2025) Allosteric regulation of UBIAD1 trafficking from ER to Golgi revealed by chemical genetic screening. Proc. Natl. Acad. Sci. USA 122: e2426532122. 40372435 10.1073/pnas.2426532122 PMC12107145

[ref58] Kanehara, K., Cho, Y., Lin, Y.C., Chen, C.E., Yu, C.Y. and Nakamura, Y. (2015) Arabidopsis DOK1 encodes a functional dolichol kinase involved in reproduction. Plant J. 81: 292–303. 25406445 10.1111/tpj.12727

[ref59] Keim, V., Manzano, D., Fernández, F.J., Closa, M., Andrade, P., Caudepón, D., et al. (2012) Characterization of *Arabidopsis* FPS isozymes and FPS gene expression analysis provide insight into the biosynthesis of isoprenoid precursors in seeds. PLoS One 7: e49109. 23145086 10.1371/journal.pone.0049109 PMC3492304

[ref60] Kopcsayova, D. and Vranova, E. (2019) Functional gene network of prenyltransferases in *Arabidopsis thaliana*. Molecules 24: 4556. 31842481 10.3390/molecules24244556 PMC6943727

[ref61] Laranjeira, S., Amorim-Silva, V., Esteban, A., Arro, M., Ferrer, A., Tavares, R.M., et al. (2015) Arabidopsis squalene epoxidase 3 (SQE3) complements SQE1 and is important for embryo development and bulk squalene epoxidase activity. Mol. Plant 8: 1090–1102. 25707755 10.1016/j.molp.2015.02.007

[ref62] Leichner, G.S., Avner, R., Harats, D. and Roitelman, J. (2011) Metabolically regulated endoplasmic reticulum-associated degradation of 3-hydroxy-3-methylglutaryl-CoA reductase: evidence for requirement of a geranylgeranylated protein. J. Biol. Chem. 286: 32150–32161. 21778231 10.1074/jbc.M111.278036 PMC3173168

[ref63] Leivar, P., Gonzalez, V.M., Castel, S., Trelease, R.N., Lopez-Iglesias, C., Arro, M., et al. (2005) Subcellular localization of *Arabidopsis* 3-hydroxy-3-methylglutaryl-coenzyme a reductase. Plant Physiol. 137: 57–69. 15618432 10.1104/pp.104.050245 PMC548838

[ref64] Li, Z.J., Wang, Y.Z., Wang, L.R., Shi, T.Q., Sun, X.M. and Huang, H. (2021) Advanced strategies for the synthesis of terpenoids in *Yarrowia lipolytica*. J. Agric. Food Chem. 69: 2367–2381. 33595318 10.1021/acs.jafc.1c00350

[ref65] Lindner, H., Kessler, S.A., Muller, L.M., Shimosato-Asano, H., Boisson-Dernier, A. and Grossniklaus, U. (2015) TURAN and EVAN mediate pollen tube reception in *Arabidopsis* synergids through protein glycosylation. PLoS Biol. 13: e1002139. 25919390 10.1371/journal.pbio.1002139 PMC4412406

[ref66] Lipko, A., Paczkowski, C., Perez-Fons, L., Fraser, P.D., Kania, M., Hoffman-Sommer, M., et al. (2023) Divergent contribution of the MVA and MEP pathways to the formation of polyprenols and dolichols in *Arabidopsis*. Biochem. J. 480: 495–520. 37022297 10.1042/BCJ20220578 PMC10212524

[ref67] Lodeiro, S., Xiong, Q., Wilson, W.K., Kolesnikova, M.D., Onak, C.S. and Matsuda, S.P. (2007) An oxidosqualene cyclase makes numerous products by diverse mechanisms: a challenge to prevailing concepts of triterpene biosynthesis. J. Am. Chem. Soc. 129: 11213–11222. 17705488 10.1021/ja073133u

[ref68] Lopez-Tubau, J.M., Laibach, N., Burciaga-Monge, A., Alseekh, S., Deng, C., Fernie, A.R., et al. (2025) Differential impact of impaired steryl ester biosynthesis on the metabolome of tomato fruits and seeds. Physiol. Plant. 177: e70022. 39710490 10.1111/ppl.70022 PMC11663625

[ref69] Lovato, M.A., Hart, E.A., Segura, M.J., Giner, J.L. and Matsuda, S.P. (2000) Functional cloning of an *Arabidopsis thaliana* cDNA encoding cycloeucalenol cycloisomerase. J. Biol. Chem. 275: 13394–13397. 10788449 10.1074/jbc.275.18.13394

[ref70] Lung, S.C., Liao, P., Yeung, E.C., Hsiao, A.S., Xue, Y. and Chye, M.L. (2017) Acyl-CoA-binding protein ACBP1 modulates sterol synthesis during embryogenesis. Plant Physiol. 174: 1420–1435. 28500265 10.1104/pp.17.00412 PMC5490911

[ref71] Luu, W., Hart-Smith, G., Sharpe, L.J. and Brown, A.J. (2015) The terminal enzymes of cholesterol synthesis, DHCR24 and DHCR7, interact physically and functionally. J. Lipid Res. 56: 888–897. 25637936 10.1194/jlr.M056986 PMC4373745

[ref72] Manzano, D., Andrade, P., Caudepon, D., Altabella, T., Arro, M. and Ferrer, A. (2016) Suppressing farnesyl diphosphate synthase alters chloroplast development and triggers sterol-dependent induction of Jasmonate- and Fe-related responses. Plant Physiol. 172: 93–117. 27382138 10.1104/pp.16.00431 PMC5074618

[ref73] Marchwicka, A., Kaminska, D., Monirialamdari, M., Blazewska, K.M. and Gendaszewska-Darmach, E. (2022) Protein prenyltransferases and their inhibitors: structural and functional characterization. Int. J. Mol. Sci. 23: 5424. 35628237 10.3390/ijms23105424 PMC9141697

[ref75] Mazein, A., Watterson, S., Hsieh, W.Y., Griffiths, W.J. and Ghazal, P. (2013) A comprehensive machine-readable view of the mammalian cholesterol biosynthesis pathway. Biochem. Pharmacol. 86: 56–66. 23583456 10.1016/j.bcp.2013.03.021 PMC3912678

[ref76] Meng, F., Zhao, Y., Titus, T., Zhang, C. and Postlethwait, J.H. (2018) Brain of the blind: transcriptomics of the golden-line cavefish brain. Curr. Zool. 64: 765–773. 30538736 10.1093/cz/zoy005 PMC6280103

[ref77] Mialoundama, A.S., Jadid, N., Brunel, J., et al. (2013) Arabidopsis ERG28 tethers the sterol C4-demethylation complex to prevent accumulation of a biosynthetic intermediate that interferes with polar auxin transport. Plant Cell 25: 4879–4893. 24326590 10.1105/tpc.113.115576 PMC3903993

[ref78] Mitsche, M.A., McDonald, J.G., Hobbs, H.H. and Cohen, J.C. (2015) Flux analysis of cholesterol biosynthesis in vivo reveals multiple tissue and cell-type specific pathways. Elife 4: e07999. 26114596 10.7554/eLife.07999 PMC4501332

[ref79] Mo, C. and Bard, M. (2005) A systematic study of yeast sterol biosynthetic protein-protein interactions using the split-ubiquitin system. Biochim. Biophys. Acta 1737: 152–160. 16300994 10.1016/j.bbalip.2005.11.002

[ref80] Mo, C., Valachovic, M., Randall, S.K., Nickels, J.T. and Bard, M. (2002) Protein-protein interactions among C-4 demethylation enzymes involved in yeast sterol biosynthesis. Proc. Natl. Acad. Sci. USA 99: 9739–9744. 12119386 10.1073/pnas.112202799 PMC124998

[ref81] Mo, C., Milla, P., Athenstaedt, K., Ott, R., Balliano, G., Daum, G., et al. (2004a) In yeast sterol biosynthesis the 3-keto reductase protein (Erg27p) is required for oxidosqualene cyclase (Erg7p) activity. Biochim. Biophys. Acta 1633: 68–74. 10.1016/s1388-1981(03)00088-x 12842197

[ref82] Mo, C., Valachovic, M. and Bard, M. (2004b) The ERG28-encoded protein, Erg28p, interacts with both the sterol C-4 demethylation enzyme complex as well as the late biosynthetic protein, the C-24 sterol methyltransferase (Erg6p). Biochim. Biophys. Acta 1686: 30–36. 15522820 10.1016/j.bbalip.2004.08.001

[ref83] Mongrand, S., Stanislas, T., Bayer, E.M., Lherminier, J. and Simon-Plas, F. (2010) Membrane rafts in plant cells. Trends Plant Sci. 15: 656–663. 20934367 10.1016/j.tplants.2010.09.003

[ref84] Morikawa, T., Mizutani, M., Aoki, N., et al. (2006) Cytochrome P450 CYP710A encodes the sterol C-22 desaturase in *Arabidopsis* and tomato. Plant Cell 18: 1008–1022. 16531502 10.1105/tpc.105.036012 PMC1425849

[ref85] Nakamoto, M., Schmit, A.C., Heintz, D., Schaller, H. and Ohta, D. (2015) Diversification of sterol methyltransferase enzymes in plants and a role for beta-sitosterol in oriented cell plate formation and polarized growth. Plant J. 84: 860–874. 26426526 10.1111/tpj.13043

[ref86] Newman, A.P. and Ferro-Novick, S. (1987) Characterization of new mutants in the early part of the yeast secretory pathway isolated by a [3H]mannose suicide selection. J. Cell Biol. 105: 1587–1594. 3312234 10.1083/jcb.105.4.1587 PMC2114650

[ref87] Ohyama, K., Suzuki, M., Masuda, K., Yoshida, S. and Muranaka, T. (2007) Chemical phenotypes of the hmg1 and hmg2 mutants of *Arabidopsis* demonstrate the in-planta role of HMG-CoA reductase in triterpene biosynthesis. Chem. Pharm. Bull. (Tokyo) 55: 1518–1521. 17917299 10.1248/cpb.55.1518

[ref88] Ohyama, K., Suzuki, M., Kikuchi, J., Saito, K. and Muranaka, T. (2009) Dual biosynthetic pathways to phytosterol via cycloartenol and lanosterol in *Arabidopsis*. Proc. Natl. Acad. Sci. USA 106: 725–730. 19139393 10.1073/pnas.0807675106 PMC2621255

[ref89] Phillips, M.A., Rasbery, J.M., Bartel, B. and Matsuda, S.P.T. (2006) Biosynthetic diversity in plant triterpene cyclization. Curr. Opin. Plant Biol. 9: 305–314. 16581287 10.1016/j.pbi.2006.03.004

[ref90] Phillips, M.A., D'Auria, J.C., Gershenzon, J. and Pichersky, E. (2008) The *Arabidopsis thaliana* type I isopentenyl diphosphate isomerases are targeted to multiple subcellular compartments and have overlapping functions in isoprenoid biosynthesis. Plant Cell 20: 677–696. 18319397 10.1105/tpc.107.053926 PMC2329938

[ref91] Pinheiro, H., Samalova, M., Geldner, N., Chory, J., Martinez, A. and Moore, I. (2009) Genetic evidence that the higher plant Rab-D1 and Rab-D2 GTPases exhibit distinct but overlapping interactions in the early secretory pathway. J. Cell Sci. 122: 3749–3758. 19789181 10.1242/jcs.050625 PMC2758805

[ref92] Pose, D., Castanedo, I., Borsani, O., Nieto, B., Rosado, A., Taconnat, L., et al. (2009) Identification of the *Arabidopsis* dry2/sqe1-5 mutant reveals a central role for sterols in drought tolerance and regulation of reactive oxygen species. Plant J. 59: 63–76. 19309460 10.1111/j.1365-313X.2009.03849.x

[ref93] Pu, X., Dong, X., Li, Q., Chen, Z. and Liu, L. (2021) An update on the function and regulation of methylerythritol phosphate and mevalonate pathways and their evolutionary dynamics. J. Integr. Plant Biol. 63: 1211–1226. 33538411 10.1111/jipb.13076

[ref94] Rahier, A. and Karst, F. (2014) Plant cyclopropylsterol-cycloisomerase: key amino acids affecting activity and substrate specificity. Biochem. J. 459: 289–299. 24483781 10.1042/BJ20131239

[ref95] Randall, S.K., Marshall, M.S. and Crowell, D.N. (1993) Protein isoprenylation in suspension-cultured tobacco cells. Plant Cell 5: 433–442. 8485402 10.1105/tpc.5.4.433 PMC160282

[ref96] Rasbery, J.M., Shan, H., LeClair, R.J., Norman, M., Matsuda, S.P. and Bartel, B. (2007) *Arabidopsis thaliana* squalene epoxidase 1 is essential for root and seed development. J. Biol. Chem. 282: 17002–17013. 17426032 10.1074/jbc.M611831200

[ref97] Rinaldi, M.A., Ferraz, C.A. and Scrutton, N.S. (2022) Alternative metabolic pathways and strategies to high-titre terpenoid production in *Escherichia coli*. Nat. Prod. Rep. 39: 90–118. 34231643 10.1039/d1np00025j PMC8791446

[ref98] Rodriguez-Concepcion, M. and Boronat, A. (2015) Breaking new ground in the regulation of the early steps of plant isoprenoid biosynthesis. Curr. Opin. Plant Biol. 25: 17–22. 25909859 10.1016/j.pbi.2015.04.001

[ref99] Rodriguez-Concepcion, M., Yalovsky, S. and Gruissem, W. (1999) Protein prenylation in plants: old friends and new targets. Plant Mol. Biol. 39: 865–870. 10344192 10.1023/a:1006170020836

[ref100] Rojek, J., Tucker, M.R., Pinto, S.C., Rychlowski, M., Lichocka, M., Soukupova, H., et al. (2021a) Rab-dependent vesicular traffic affects female gametophyte development in Arabidopsis. J. Exp. Bot. 72: 320–340. 32939545 10.1093/jxb/eraa430 PMC7853608

[ref101] Rojek, J., Tucker, M.R., Rychlowski, M., Nowakowska, J. and Gutkowska, M. (2021b) The Rab geranylgeranyl transferase Beta subunit is essential for embryo and seed development in *Arabidopsis thaliana*. Int. J. Mol. Sci. 22: 7907. 34360673 10.3390/ijms22157907 PMC8347404

[ref102] Ruiz-Sola, M.A., Barja, M.V., Manzano, D., Llorente, B., Schipper, B., Beekwilder, J., et al. (2016a) A single *Arabidopsis* gene encodes two differentially targeted geranylgeranyl diphosphate synthase isoforms. Plant Physiol. 172: 1393–1402. 27707890 10.1104/pp.16.01392 PMC5100792

[ref103] Ruiz-Sola, M.A., Coman, D., Beck, G., et al. (2016b) Arabidopsis geranylgeranyl diphosphate synthase 11 is a hub isozyme required for the production of most photosynthesis-related isoprenoids. New Phytol. 209: 252–264. 26224411 10.1111/nph.13580

[ref104] Running, M.P. (2014) The role of lipid post-translational modification in plant developmental processes. Front. Plant Sci. 5: 50. 24600462 10.3389/fpls.2014.00050 PMC3927097

[ref105] Running, M.P., Lavy, M., Sternberg, H., Galichet, A., Gruissem, W., Hake, S., et al. (2004) Enlarged meristems and delayed growth in plp mutants result from lack of CaaX prenyltransferases. Proc. Natl. Acad. Sci. USA 101: 7815–7820. 15128936 10.1073/pnas.0402385101 PMC419689

[ref106] Schaeffer, A., Bronner, R., Benveniste, P. and Schaller, H. (2001) The ratio of campesterol to sitosterol that modulates growth in Arabidopsis is controlled by sterol methyltransferase 2;1. Plant J. 25: 605–615. 11319028 10.1046/j.1365-313x.2001.00994.x

[ref107] Segura, M.J., Meyer, M.M. and Matsuda, S.P. (2000) *Arabidopsis thaliana* LUP1 converts oxidosqualene to multiple triterpene alcohols and a triterpene diol. Org. Lett. 2: 2257–2259. 10930257 10.1021/ol006016b

[ref108] Sharpe, L.J., Coates, H.W. and Brown, A.J. (2020) Post-translational control of the long and winding road to cholesterol. J. Biol. Chem. 295: 17549–17559. 33453997 10.1074/jbc.REV120.010723 PMC7762936

[ref109] Shi, W., Zeng, Q., Kunkel, B.N. and Running, M.P. (2016) Arabidopsis Rab geranylgeranyltransferases demonstrate redundancy and broad substrate specificity in vitro. J. Biol. Chem. 291: 1398–1410. 26589801 10.1074/jbc.M115.673491 PMC4714223

[ref110] Shibuya, M., Katsube, Y., Otsuka, M., Zhang, H., Tansakul, P., Xiang, T., et al. (2009) Identification of a product specific beta-amyrin synthase from *Arabidopsis thaliana*. Plant Physiol. Biochem. 47: 26–30. 18977664 10.1016/j.plaphy.2008.09.007

[ref111] Shimada, T.L., Shimada, T., Okazaki, Y., et al. (2019) High sterol ester 1 is a key factor in plant sterol homeostasis. Nat. Plants 5: 1154–1166. 31712757 10.1038/s41477-019-0537-2

[ref112] Shipton, C.A., Parmryd, I., Swiezewska, E., Andersson, B. and Dallner, G. (1995) Isoprenylation of plant proteins in vivo. Isoprenylated proteins are abundant in the mitochondria and nuclei of spinach. J. Biol. Chem. 270: 566–572. 7822281 10.1074/jbc.270.2.566

[ref113] Shirakawa, R., Goto-Ito, S., Goto, K., et al. (2020) A SNARE geranylgeranyltransferase essential for the organization of the Golgi apparatus. EMBO J. 39: e104120. 32128853 10.15252/embj.2019104120 PMC7156963

[ref114] Sonawane, P.D., Pollier, J., Panda, S., et al. (2016) Plant cholesterol biosynthetic pathway overlaps with phytosterol metabolism. Nat. Plants 3: 16205. 28005066 10.1038/nplants.2016.205

[ref115] Song, B.L., Javitt, N.B. and DeBose-Boyd, R.A. (2005) Insig-mediated degradation of HMG CoA reductase stimulated by lanosterol, an intermediate in the synthesis of cholesterol. Cell Metab. 1: 179–189. 16054061 10.1016/j.cmet.2005.01.001

[ref116] Song, J., Sun, S., Ren, H., Grison, M., Boutte, Y., Bai, W., et al. (2019) The SMO1 family of sterol 4alpha-methyl oxidases is essential for auxin- and cytokinin-regulated embryogenesis. Plant Physiol. 181: 578–594. 31341004 10.1104/pp.19.00144 PMC6776873

[ref117] Surmacz, L. and Swiezewska, E. (2011) Polyisoprenoids - secondary metabolites or physiologically important superlipids? Biochem. Biophys. Res. Commun. 407: 627–632. 21419101 10.1016/j.bbrc.2011.03.059

[ref118] Suzuki, M., Xiang, T., Ohyama, K., et al. (2006) Lanosterol synthase in dicotyledonous plants. Plant Cell Physiol. 47: 565–571. 16531458 10.1093/pcp/pcj031

[ref119] Suzuki, M., Nakagawa, S., Kamide, Y., Kobayashi, K., Ohyama, K., Hashinokuchi, H., et al. (2009) Complete blockage of the mevalonate pathway results in male gametophyte lethality. J. Exp. Bot. 60: 2055–2064. 19363204 10.1093/jxb/erp073 PMC2682496

[ref120] Swiezewska, E., Thelin, A., Dallner, G., Andersson, B. and Ernster, L. (1993) Occurrence of prenylated proteins in plant cells. Biochem. Biophys. Res. Commun. 192: 161–166. 8476417 10.1006/bbrc.1993.1395

[ref121] Tateishi, M., Goto, K., Hishinuma, E., Matsukawa, N., Kishimoto, T., Tanaka, K., et al. (2025) Double prenylation of budding yeast Ykt6 regulates cell wall integrity and autophagy. J. Biol. Chem. 301: 108384. 40049413 10.1016/j.jbc.2025.108384 PMC12001115

[ref122] Theesfeld, C.L. and Hampton, R.Y. (2013) Insulin-induced gene protein (INSIG)-dependent sterol regulation of Hmg2 endoplasmic reticulum-associated degradation (ERAD) in yeast. J. Biol. Chem. 288: 8519–8530. 23306196 10.1074/jbc.M112.404517 PMC3605666

[ref123] Turnbull, D. and Hemsley, P.A. (2017) Fats and function: protein lipid modifications in plant cell signalling. Curr. Opin. Plant Biol. 40: 63–70. 28772175 10.1016/j.pbi.2017.07.007

[ref124] Valtersson, C., van Duyn, G., Verkleij, A.J., Chojnacki, T., de Kruijff, B. and Dallner, G. (1985) The influence of dolichol, dolichol esters, and dolichyl phosphate on phospholipid polymorphism and fluidity in model membranes. J. Biol. Chem. 260: 2742–2751. 3919007

[ref125] Van Gelder, K., Virta, L.K.A., Easlick, J., Prudhomme, N., McAlister, J.A., Geddes-McAlister, J., et al. (2021) A central role for polyprenol reductase in plant dolichol biosynthesis. Plant Sci. 303: 110773. 33487357 10.1016/j.plantsci.2020.110773

[ref126] Villette, C., Berna, A., Compagnon, V. and Schaller, H. (2015) Plant sterol diversity in pollen from angiosperms. Lipids 50: 749–760. 25820807 10.1007/s11745-015-4008-x

[ref127] Vranova, E., Hirsch-Hoffmann, M. and Gruissem, W. (2011) AtIPD: a curated database of *Arabidopsis* isoprenoid pathway models and genes for isoprenoid network analysis. Plant Physiol. 156: 1655–1660. 21617028 10.1104/pp.111.177758 PMC3149963

[ref128] Wang, M. and Casey, P.J. (2016) Protein prenylation: unique fats make their mark on biology. Nat. Rev. Mol. Cell Biol. 17: 110–122. 26790532 10.1038/nrm.2015.11

[ref129] Wang, Z., Nelson, D.R., Zhang, J., Wan, X. and Peters, R.J. (2023) Plant (di)terpenoid evolution: from pigments to hormones and beyond. Nat. Prod. Rep. 40: 452–469. 36472136 10.1039/d2np00054g PMC9945934

[ref130] Wangeline, M.A. and Hampton, R.Y. (2018) “Mallostery”-ligand-dependent protein misfolding enables physiological regulation by ERAD. J. Biol. Chem. 293: 14937–14950. 30018140 10.1074/jbc.RA118.001808 PMC6153289

[ref131] Wangeline, M.A. and Hampton, R.Y. (2021) An autonomous, but INSIG-modulated, role for the sterol sensing domain in mallostery-regulated ERAD of yeast HMG-CoA reductase. J. Biol. Chem. 296: 100063. 33184059 10.1074/jbc.RA120.015910 PMC7948459

[ref132] Warchol, I., Gora, M., Wysocka-Kapcinska, M., et al. (2016) Genetic engineering and molecular characterization of yeast strain expressing hybrid human-yeast squalene synthase as a tool for anti-cholesterol drug assessment. J. Appl. Microbiol. 120: 877–888. 26757023 10.1111/jam.13053

[ref133] Weimer, M.R., Balke, N.E. and Buhler, D.D. (1992) Herbicide clomazone does not inhibit in vitro geranylgeranyl synthesis from mevalonate. Plant Physiol. 98: 427–432. 16668657 10.1104/pp.98.2.427 PMC1080206

[ref134] Wentzinger, L.F., Bach, T.J. and Hartmann, M.A. (2002) Inhibition of squalene synthase and squalene epoxidase in tobacco cells triggers an up-regulation of 3-hydroxy-3-methylglutaryl coenzyme a reductase. Plant Physiol. 130: 334–346. 12226513 10.1104/pp.004655 PMC166566

[ref135] Wille, A., Zimmermann, P., Vranova, E., et al. (2004) Sparse graphical gaussian modeling of the isoprenoid gene network in *Arabidopsis thaliana*. Genome Biol. 5: R92. 15535868 10.1186/gb-2004-5-11-r92 PMC545783

[ref136] Willemsen, V., Friml, J., Grebe, M., van den Toorn, A., Palme, K. and Scheres, B. (2003) Cell polarity and PIN protein positioning in Arabidopsis require sterol methyltransferase1 function. Plant Cell 15: 612–625. 12615936 10.1105/tpc.008433 PMC150017

[ref137] Xue, Z., Duan, L., Liu, D., Guo, J., Ge, S., Dicks, J., et al. (2012) Divergent evolution of oxidosqualene cyclases in plants. New Phytol. 193: 1022–1038. 22150097 10.1111/j.1469-8137.2011.03997.x

[ref138] Youn, J.H., Kim, T.W., Joo, S.H., Son, S.H., Roh, J., Kim, S., et al. (2018) Function and molecular regulation of DWARF1 as a C-24 reductase in brassinosteroid biosynthesis in Arabidopsis. J. Exp. Bot. 69: 1873–1886. 29432595 10.1093/jxb/ery038 PMC6018864

[ref139] Yu, L., Fan, J., Zhou, C. and Xu, C. (2021) Sterols are required for the coordinated assembly of lipid droplets in developing seeds. Nat. Commun. 12: 5598. 34552075 10.1038/s41467-021-25908-6 PMC8458542

[ref140] Zhang, H., Ohyama, K., Boudet, J., Chen, Z., Yang, J., Zhang, M., et al. (2008) Dolichol biosynthesis and its effects on the unfolded protein response and abiotic stress resistance in *Arabidopsis*. Plant Cell 20: 1879–1898. 18612099 10.1105/tpc.108.061150 PMC2518237

[ref141] Zheng, H., Camacho, L., Wee, E., Batoko, H., Legen, J., Leaver, C.J., et al. (2005) A Rab-E GTPase mutant acts downstream of the Rab-D subclass in biosynthetic membrane traffic to the plasma membrane in tobacco leaf epidermis. Plant Cell 17: 2020–2036. 15972698 10.1105/tpc.105.031112 PMC1167549

[ref142] Zhou, X., Rao, S., Wrightstone, E., Sun, T., Lui, A.C.W., Welsch, R., et al. (2022) Phytoene synthase: the key rate-limiting enzyme of carotenoid biosynthesis in plants. Front. Plant Sci. 13: 884720. 35498681 10.3389/fpls.2022.884720 PMC9039723

[ref143] Zu, P., Koch, H., Schwery, O., et al. (2021) Pollen sterols are associated with phylogeny and environment but not with pollinator guilds. New Phytol. 230: 1169–1184. 33484583 10.1111/nph.17227 PMC8653887

