# Supplementary figures and images for: Rab geranylgeranyl transferase activity is required for proper sterol biosynthesis in Arabidopsis thaliana
Source: Plant Cell Physiol. 2025 Dec 10;67(3):346–66. doi: 10.1093/pcp/pcaf166 (PMC13078166; doi:10.1093/pcp/pcaf166)

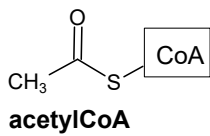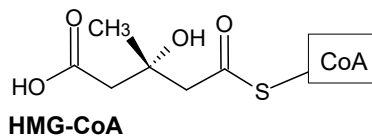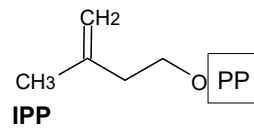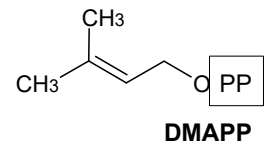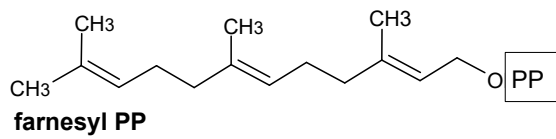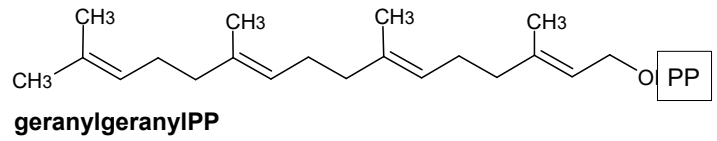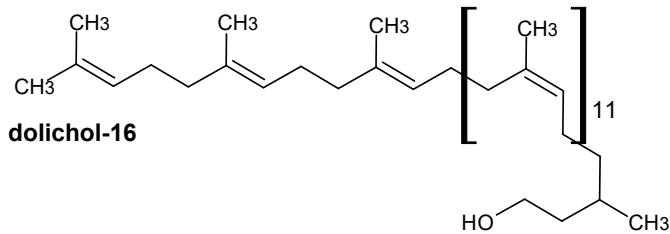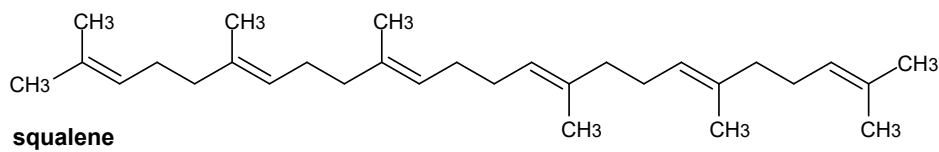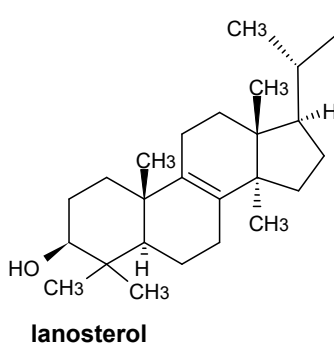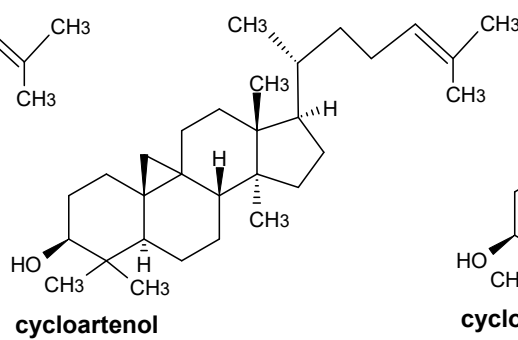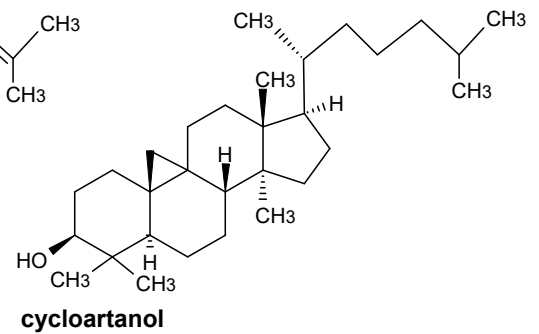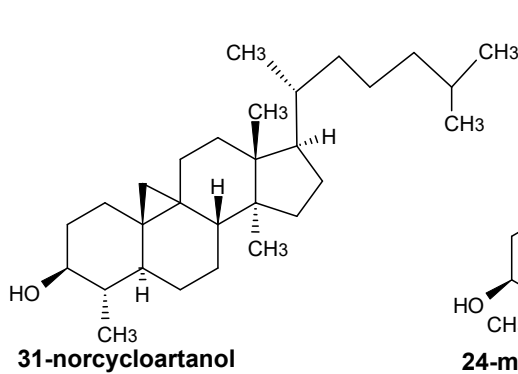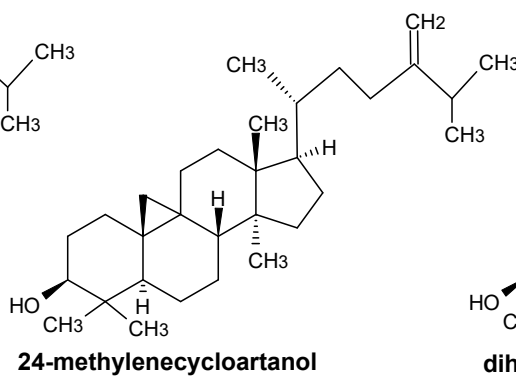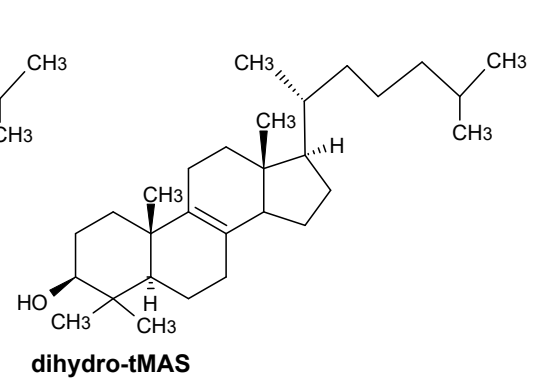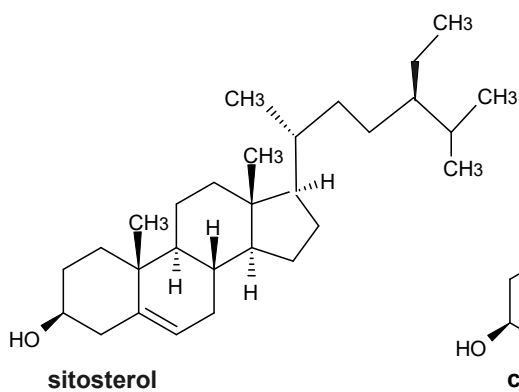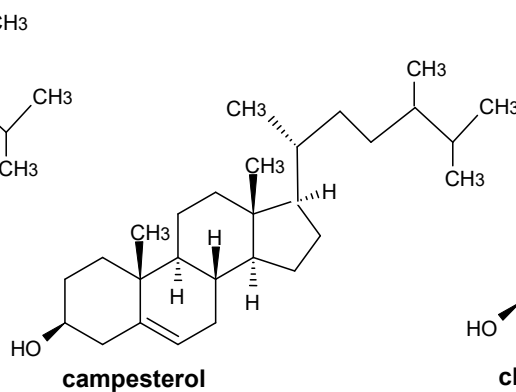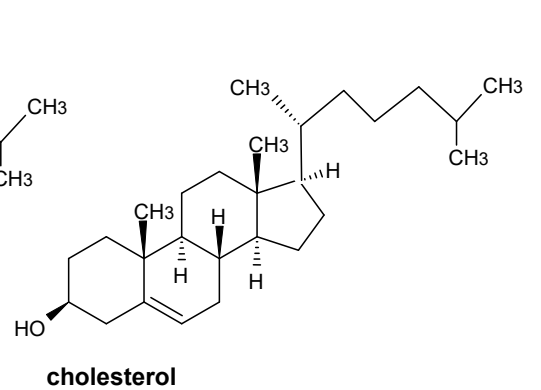

Supplement: SuppFig1_Chemical_formulas_pcaf166 [file suppfig1_chemical_formulas_pcaf166.pdf]

WT

rgtb1-2

standards mix

blank sample

dihydro-FF-MAS

dihydro-T-MAS

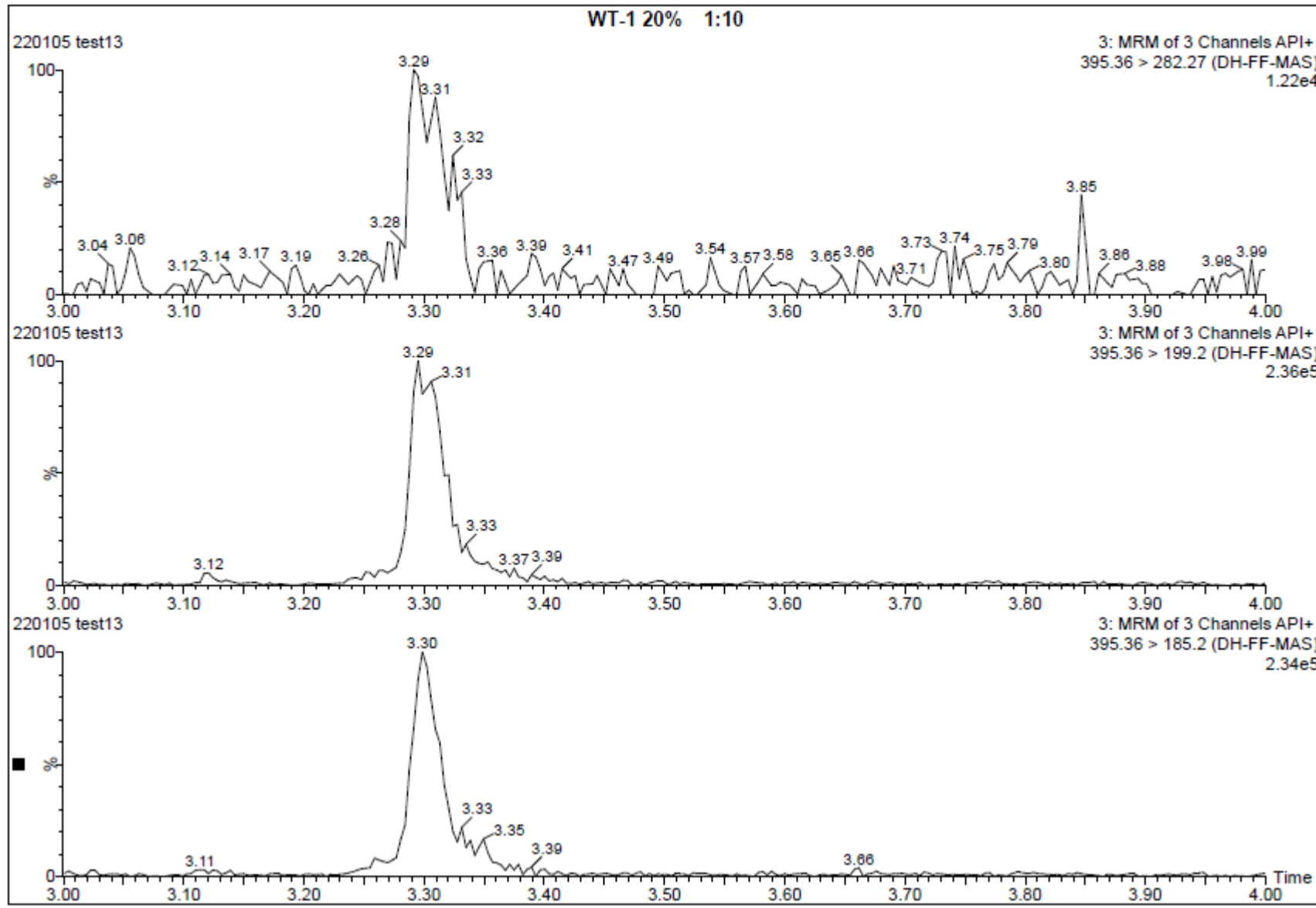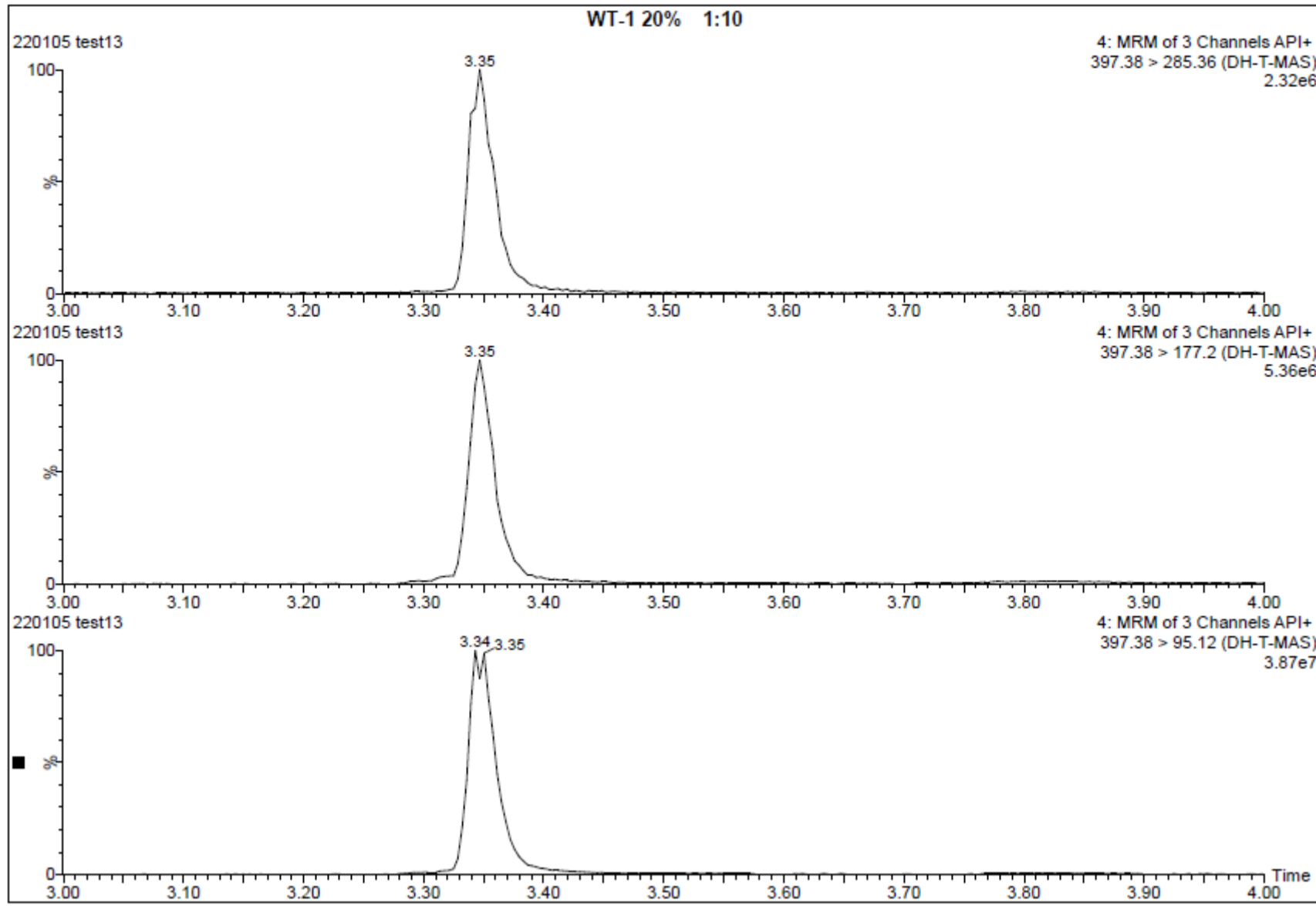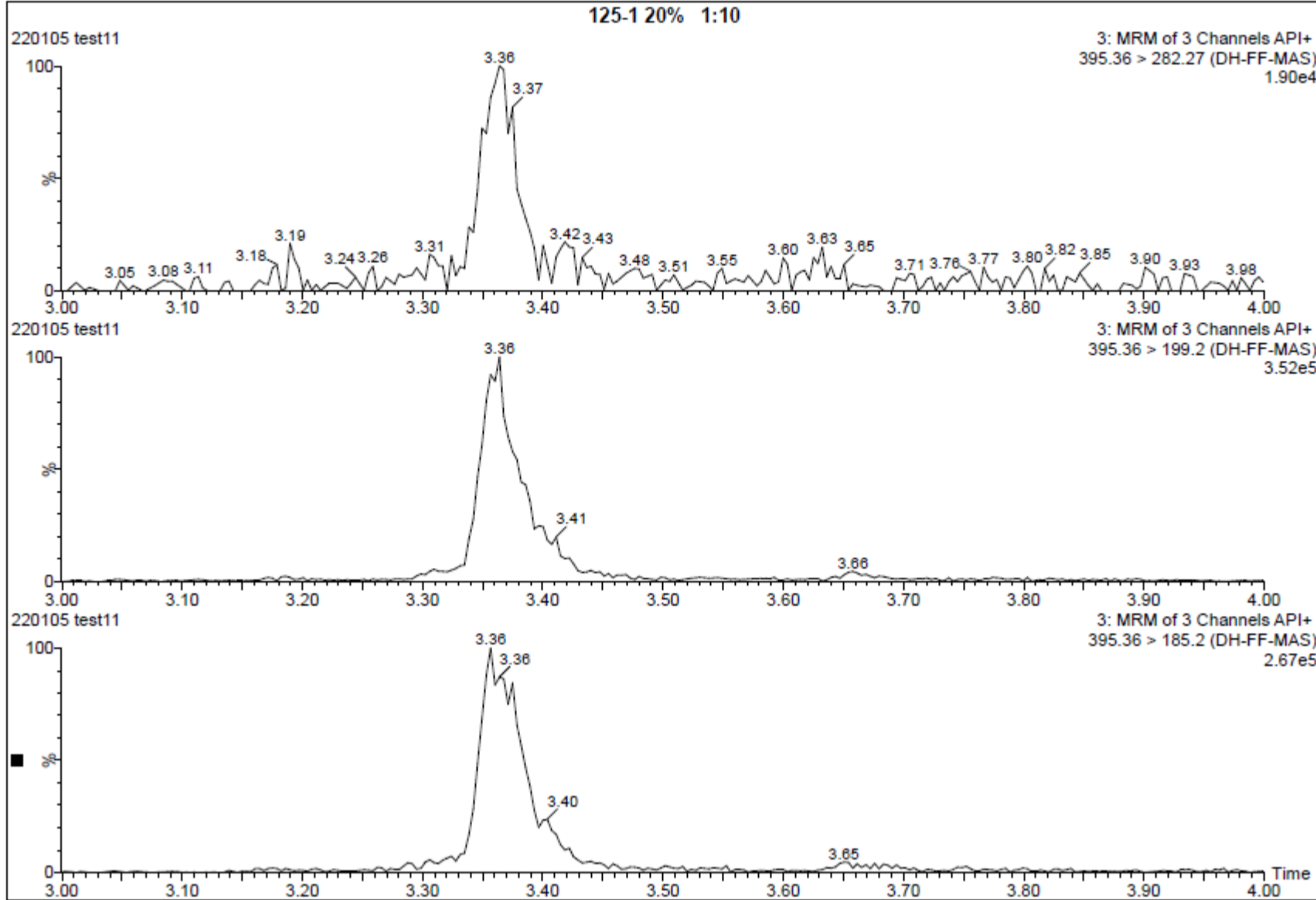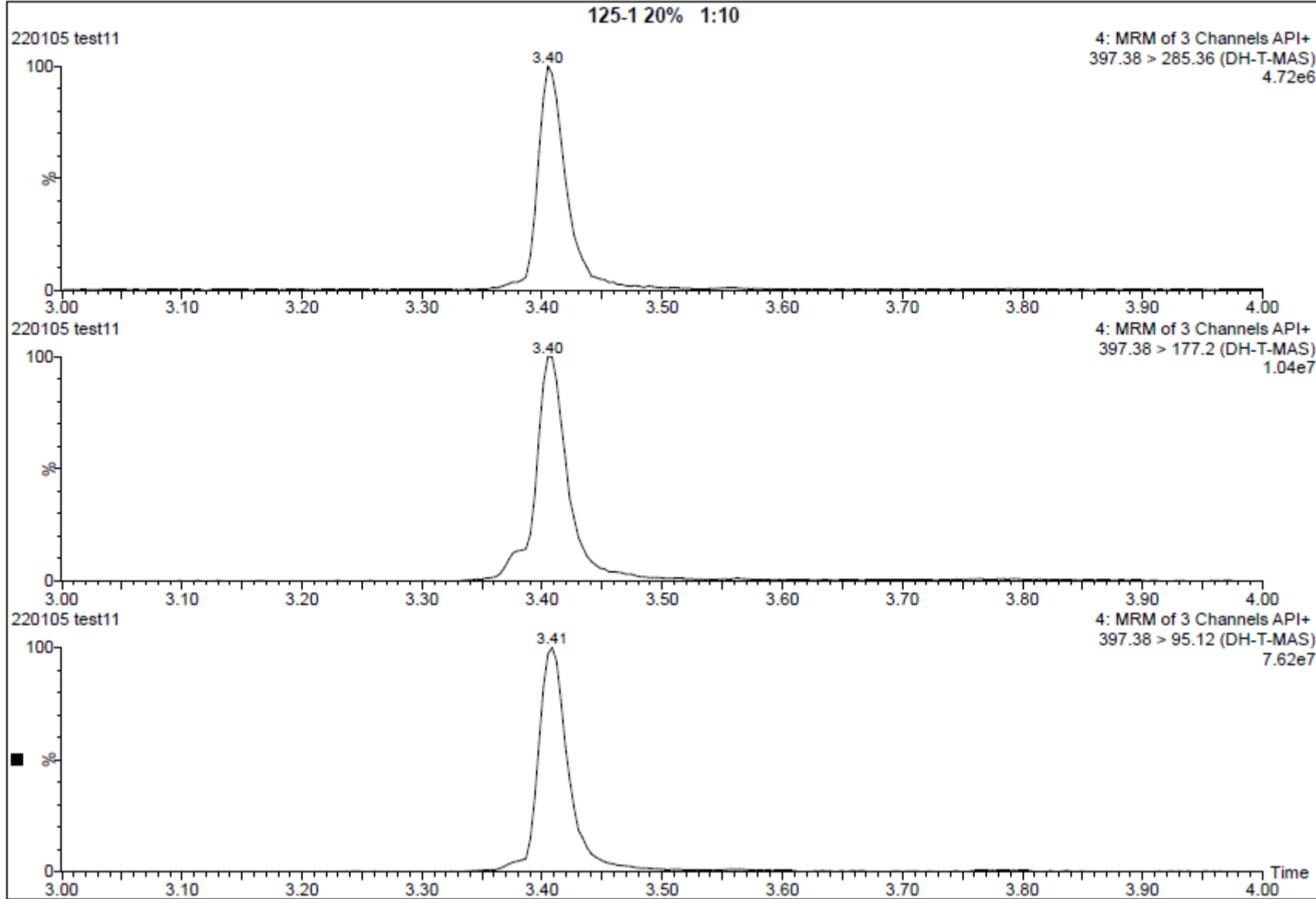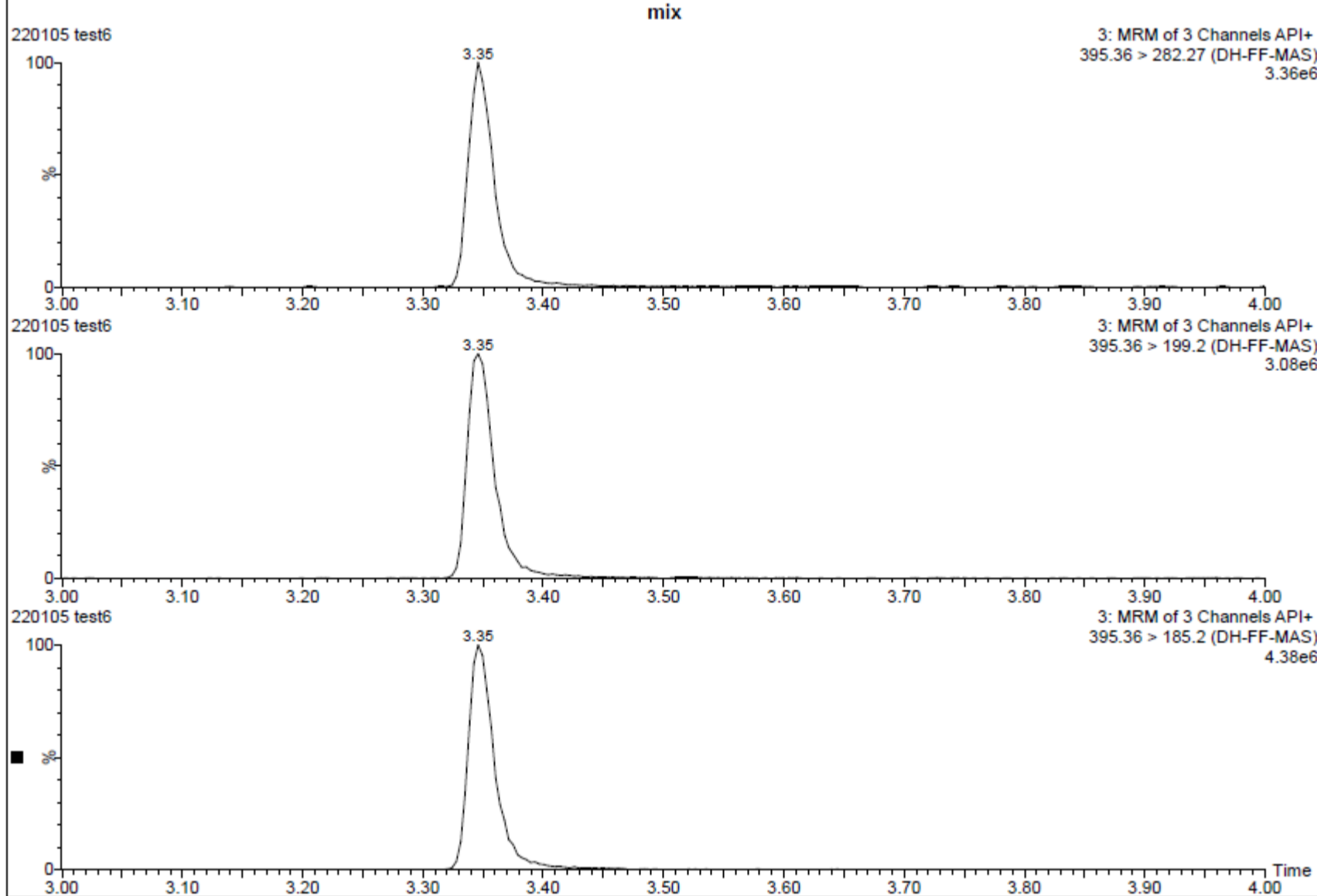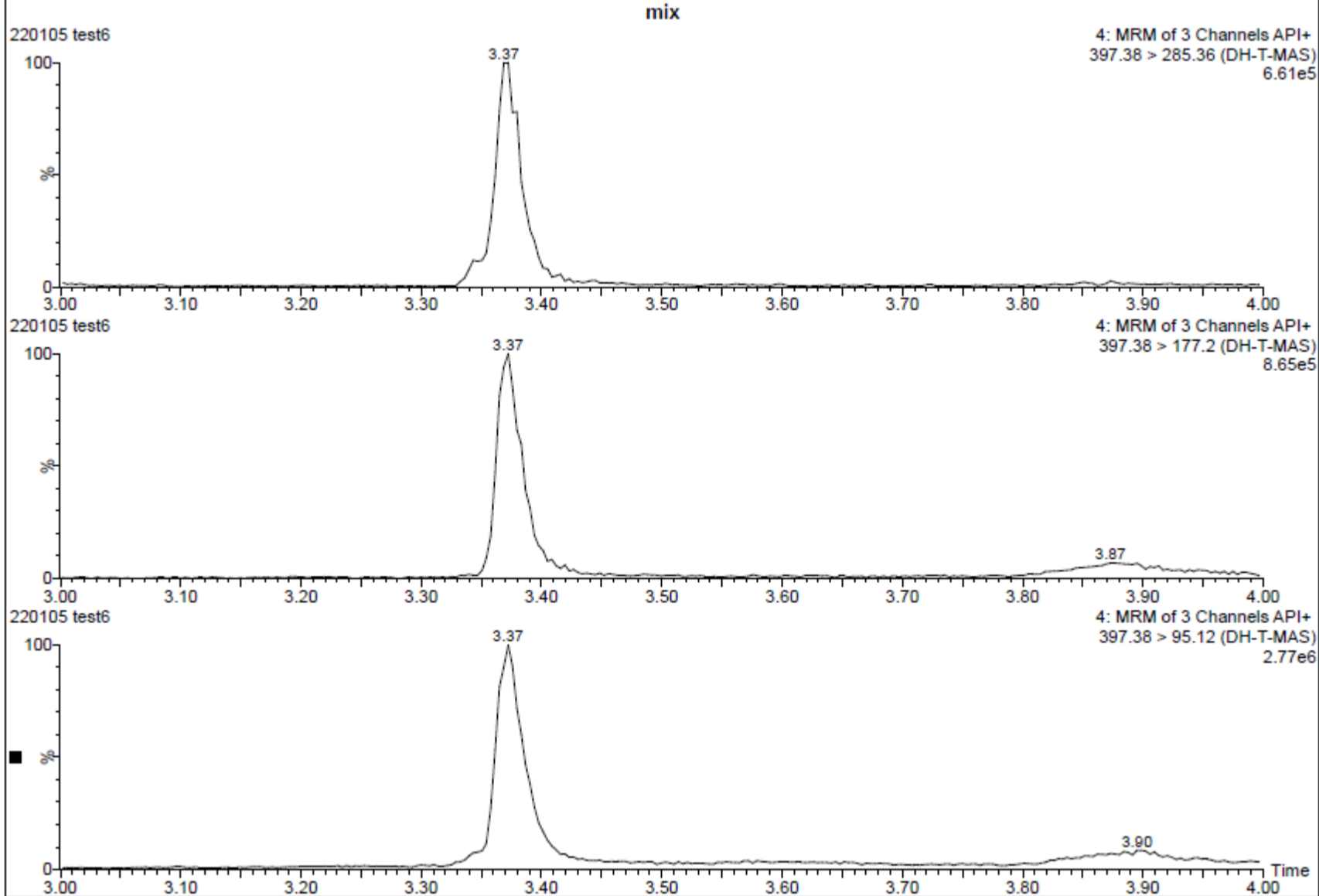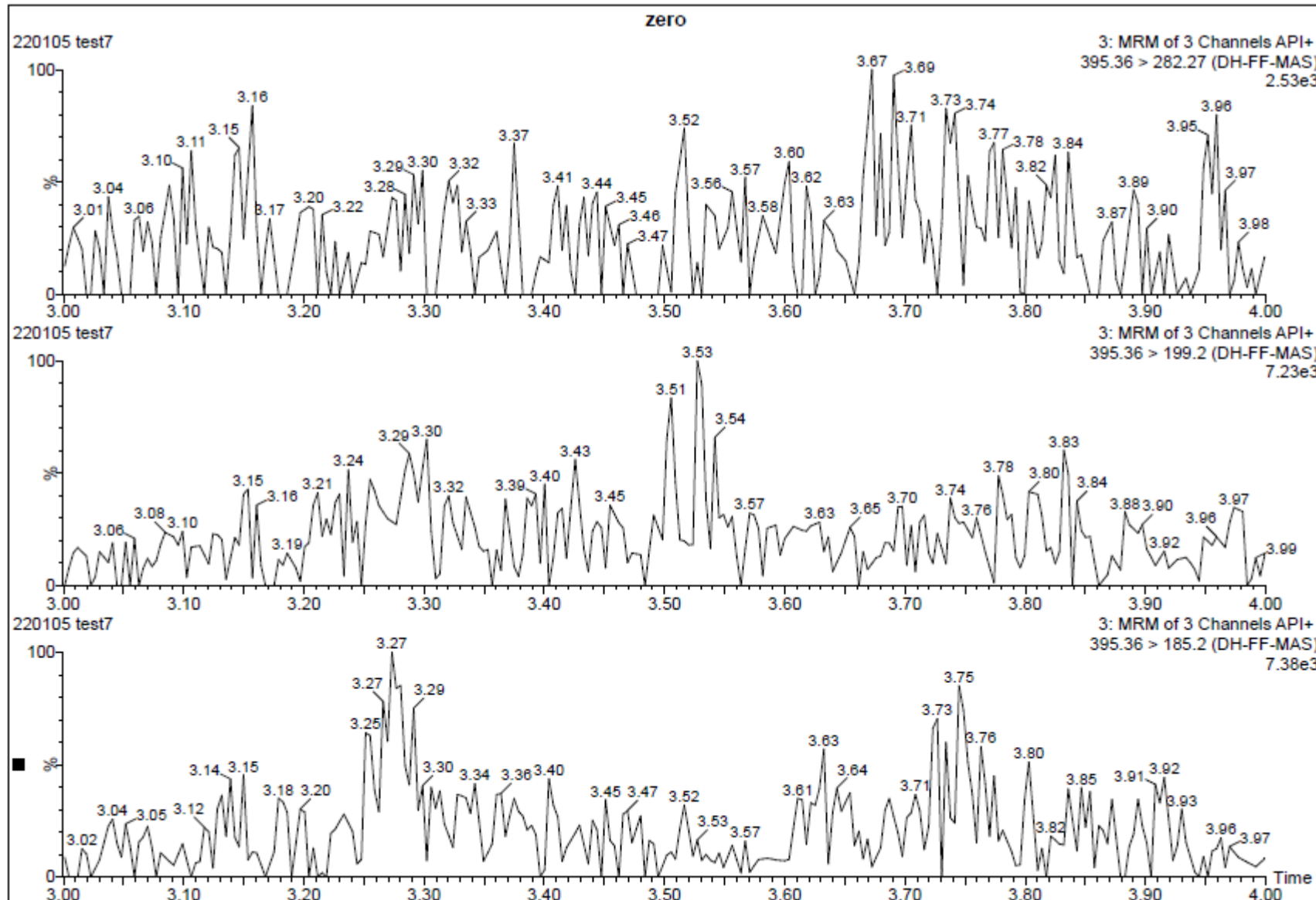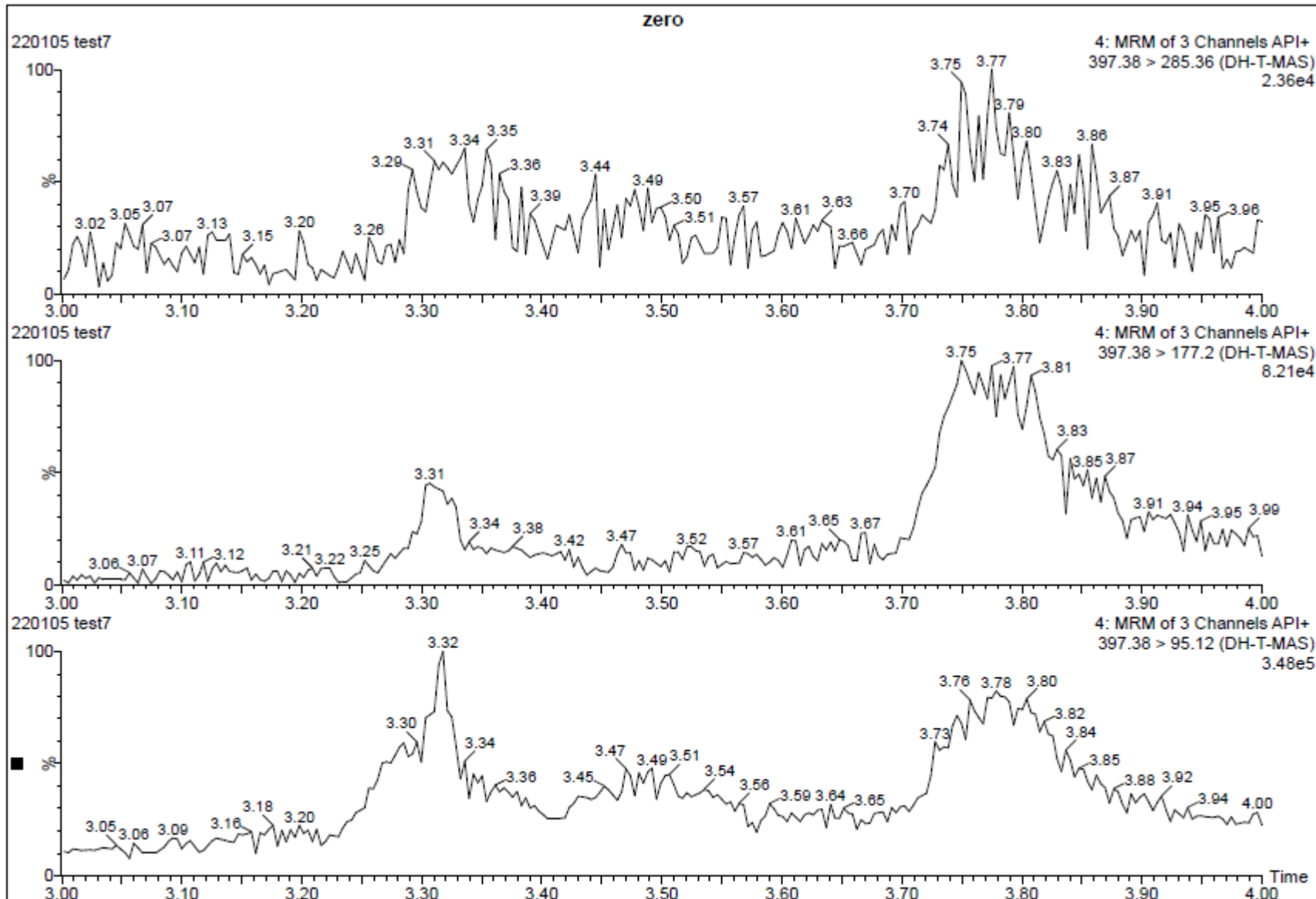

Supplement: SuppFig2_LC-MS-MRS_pcaf166 [file suppfig2_lc-ms-mrs_pcaf166.pdf]

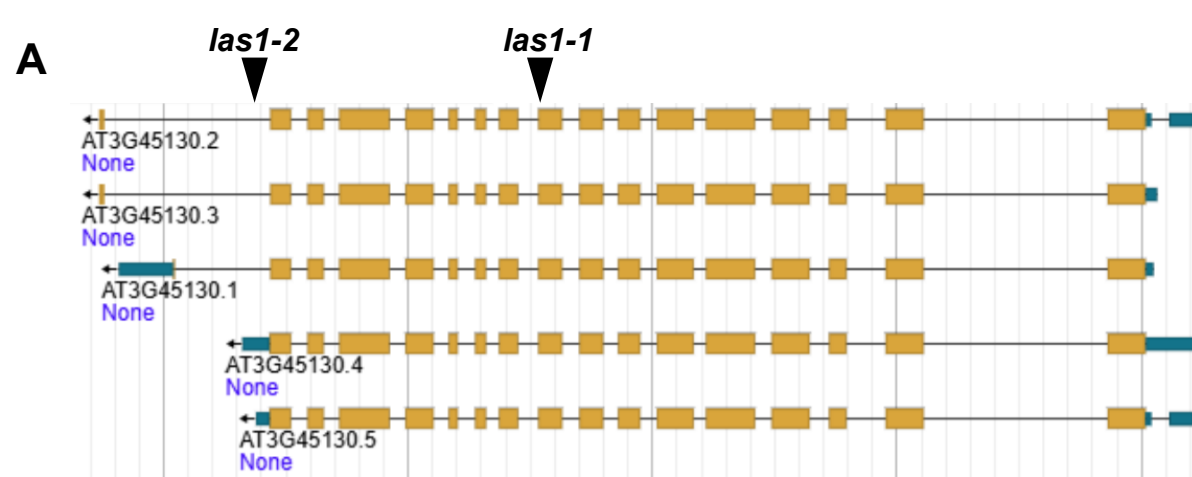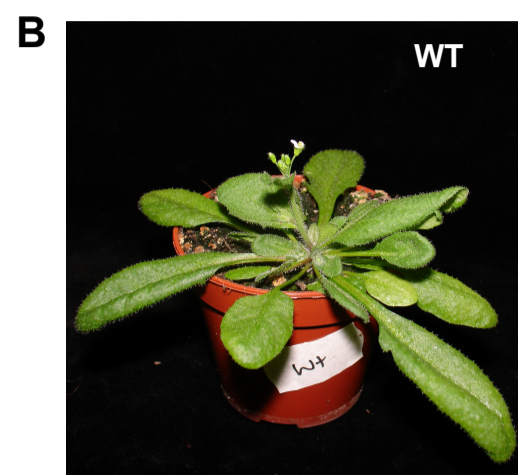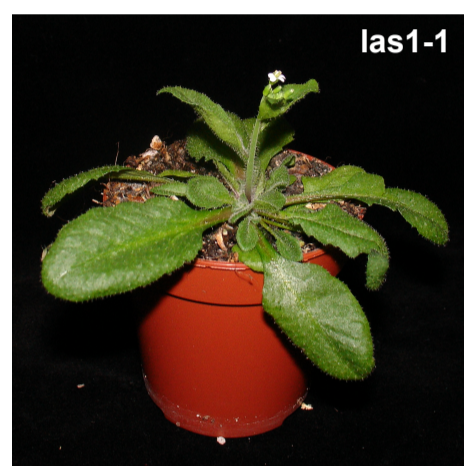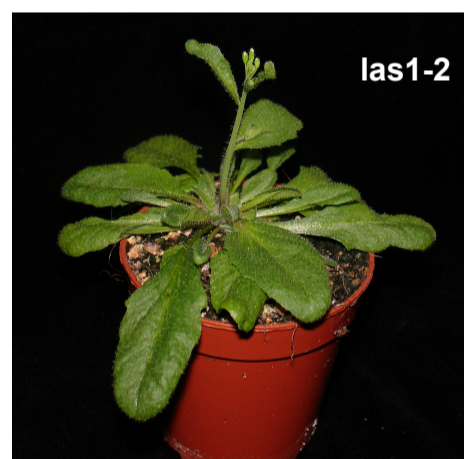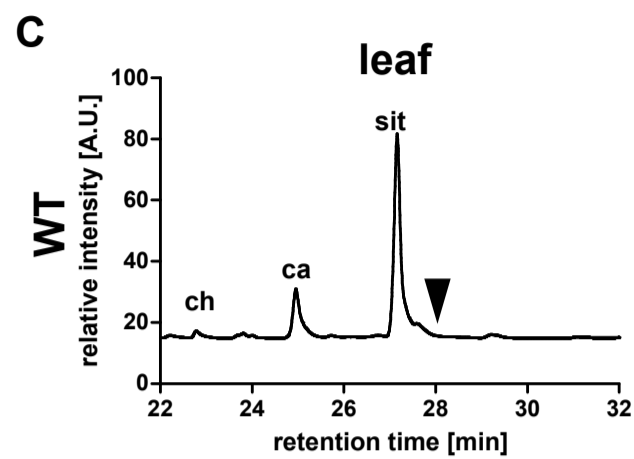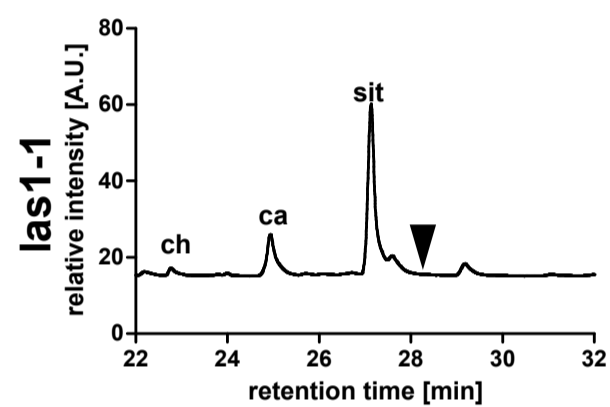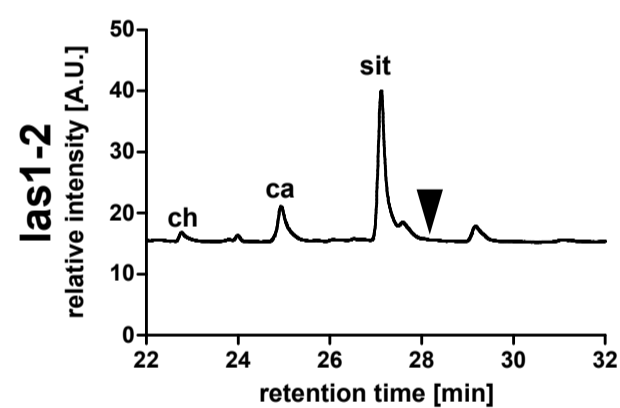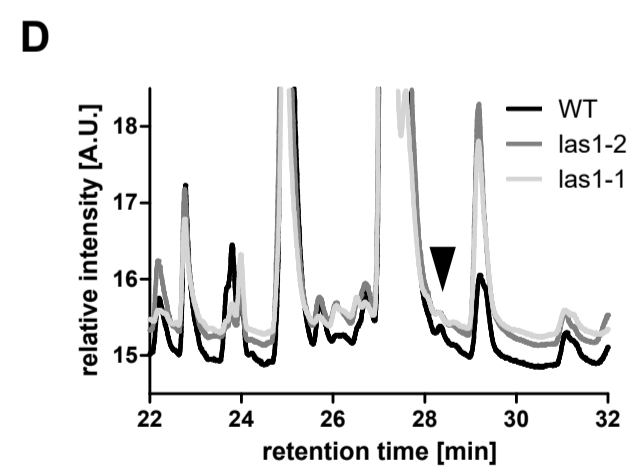

Segregation analysis:

*las1-1* (SALK 128191C)  
homozygous line

*las1-2* (SALK 058342)  
homozygotes 9 out of 39 plants

Supplement: SuppFig3_las1_mutant_pcaf166 [file suppfig3_las1_mutant_pcaf166.pdf]

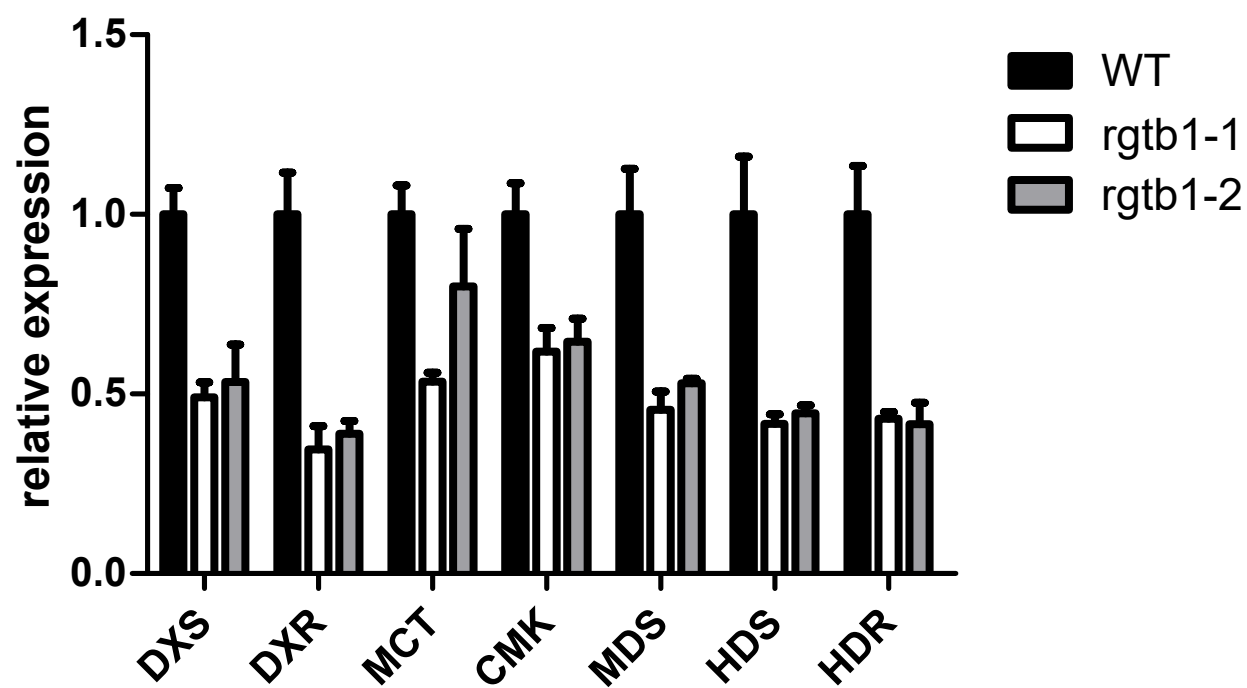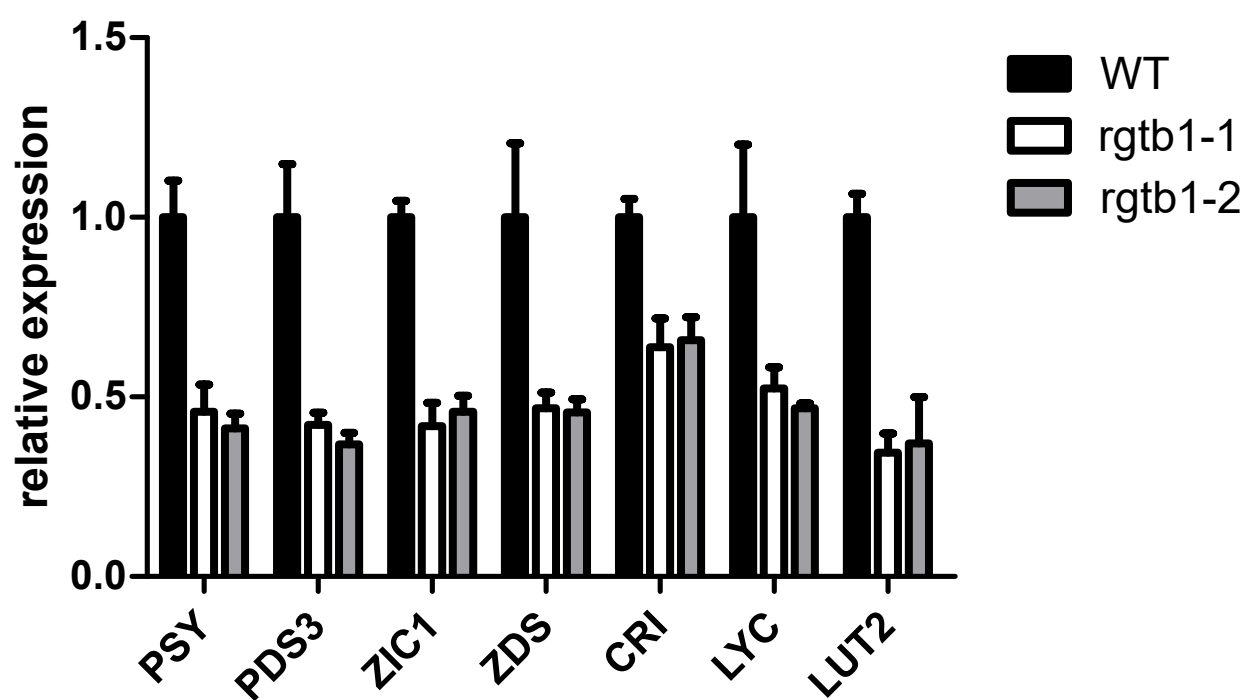

Supplement: SuppFig4_MEP_carotenoid_expression_pcaf166 [file suppfig4_mep_carotenoid_expression_pcaf166.pdf]
