# Supplementary material for: Rab geranylgeranyl transferase activity is required for proper sterol biosynthesis in Arabidopsis thaliana
Source: Plant Cell Physiol. 2025 Dec 10;67(3):346–66. doi: 10.1093/pcp/pcaf166 (PMC13078166; doi:10.1093/pcp/pcaf166)
Supplement: Table_S1_names_of_genes_MEP_and_carotenoid_pcaf166 [file table_s1_names_of_genes_mep_and_carotenoid_pcaf166.pdf]

**gene name**	**enzymatic activity**	**E.C. number**	**Arabidopsis genetic locus number**
		**MEP pathway**	
DXS	1-deoxy-D-xylulose-5-phosphate synthase	EC 2.2.1.7	At4g15560
DXR	1-deoxy-D-xylulose 5-phosphate reductoisomerase	EC 1.1.1.267	At5g62790
MCT	2-C-methyl-D-erythritol 4-phosphate cytidylyltransferase	EC 2.7.7.60	At2g02500
CMK	4-(cytidine 5'-diphospho)-2-C-methyl-D-erythritol kinase	EC 2.7.1.148	AT2G26930
MDS	2-C-methyl-D-erythritol 2,4-cyclodiphosphate synthase	EC 4.6.1.12	At1g63970
HDS	4-hydroxy-3-methylbut-2-enyl diphosphate synthase	EC 3.20.20.20	At5g60600
HDR	4-hydroxy-3-methylbut-2-enyl diphosphate reductase	EC 1.17.7.4	At4g34350
		**carotenoid biosynthesis pathway**	
PSY	phytoene synthase	EC 2.5.1.32	At5g17230
PDS3	phytoene desaturase	EC 1.3.5.5	At4g14210
ZIC1	15-cis-zeta-carotene isomerase	EC 5.2.1.12	At1g10830
ZDS	zeta-carotene desaturase	EC 1.3.5.6	At3g04870
CRI	carotenoid isomerase	EC 5.2.1.13	AT1G06820
LYC	llycopene beta-cyclase	EC 5.5.1.19	At2g32640
LUT2	Lycopene epsilon cyclase	EC 5.5.1.18	At5g57030
