# Supplementary material for: Rab geranylgeranyl transferase activity is required for proper sterol biosynthesis in Arabidopsis thaliana
Source: Plant Cell Physiol. 2025 Dec 10;67(3):346–66. doi: 10.1093/pcp/pcaf166 (PMC13078166; doi:10.1093/pcp/pcaf166)
Supplement: TableS2_primers_pcaf166 [file tables2_primers_pcaf166.pdf]

Table S2

Sequences of the primers used in this study

gene	forward primer	reverse primer
**control primer**		
PP2AA3	TAACGTGGCCAAAATGATGC	GTTCTCCACAACCGCTTGGT
**sterol biosynthesis**		
SQS1	TCGATGCTCCACAAGGTTTCT	CAAGAGCTCGGAGAACCAAG
SQE1	AGACCGCTGCGTGATCTTAG	GAGCATTCGCAAGGGTGTTG
SQE2	TTGATTCGGATGTAGCCGGG	TTCAAGCAACGACGTAACCG
SQE3	TTCGCAGTTGCGATTTTCGG	TGCCTCACACCTTCTGCTTT
CAS1	GGGTCCCATAACGTCCACTG	TTGCACAAGTGGATGTGGGT
SMT1	CTGGGATTGACGGCTTGTGA	GACGCGAGATCCATAGCACT
SMT2	AGGAGAAGGATCTGGCGAGT	TTAGGAGCAACTCCAACCGC
SMT3	GGCCCAAGTCACTGGAATCA	CCACAAACGACGTTGCAGAG
CPI1	TGCTGCTATGTTGGGAATTGAC	GCCTTAGAAACATGCCCTGC
CYP51G2	AACTTCTCCTGCATTCGCCA	GCCTACCAGCTCCCAAAGAG
HYD2	TTAGCATTCAGGGCTGGTGG	TGTTAGCTCCTCGAAAAACCA
HYD1	ACCTCGGTTCTTCCCTCCTT	ACCAGTGAATGTCCACCAACA
DWF7	AAATTACCTCGCCGGAACCC	ACATTTGCAAACGCATAGCCT
DWF5	GTTCAGGAGGACAAACGGGA	CCAATCCCCACCATCCAGAC
DWF1	TACAAGGTTGGCGATGCACT	TTTGGCCTTTGATTGGCTGC
CYP710A1	TGGCTCTCCCCATAGACCTC	TGCCATCCTCGCTTTGGATT
CYP710A2	TTTGCGTTCAACAAGGCTCG	AAGCATGTTGGCTCCTCTCC
SMO1-1	GCAACTTCGCTTCAGTGTTCA	TCGACGACTCCTTGATCTGC
SMO1-2	GTTGGAGGACAAAGCCAGAGT	CAGCTTGTTGCTCTTCTTGGA
SMO1-3	CCTATCGGATACGCATCGCC	GGGTCTCAATCGCCTCGAAT
SMO2-1	CCTCCTACCCTGTCTTCCGA	GCAAGATCCGATGACCCCAA
SMO2-2	GGGTCACAGGATCTTGCATACT	CGGACCAACAATGGTAGCAAA
DWF6	ACTTCCACCGCACCATCATT	GTAATGCGAAACCCACCTCG
3BHSDD1	AGCTCAAAATCAACCCAAAACAGA	CCGGTGGTTGCAACAAAGTT
ERG28	TGCGTTAGGCCATTTTCTGAC	AGTGCAGGTCAAGAGTGTCC
**MVA pathway**		
AACT1	CCTCATCTGCCACCAAACCA	AAAGACGAGAGGGAGCCAAG
AACT2	AAAGCCATTGCACATGCTGG	CTGGAGCAATCCCGAGTAGC
HMGS	ACGAGAGTTACCAAAGCCGT	GGGAAGCAAATGCAGCGTAG
HMGR1	TCTATCGAGGTGGGGACAGT	GATCGTCGCTAGCCTCCTTG
HMGR2	CCCAGCTCAAAACGTGGAGA	TCCCACCTCCAACAGTACCA
MK	ATGTGGCTCTCCTCTGGGAT	CCAGACCCGTATGGAAGCTC
PMK	TATGCGTCAGATGGGTGAGG	CTCCAGGAACACCAGCAAGT
MPDC1	CAGTTGAATTGCTGCAGGGG	CCTTCCAAGCCTGCCTCTTT
MPDC2	TGGAACCGTTCTGAAGGGAC	GCAATTGGACTGCGACCTTT
**short isoprenoids biosynthesis**		
IPPI1	GTGCTTTCTCTGGTACCGCT	TACTTGCTGTCATGCCCCAC
IPPI2	AAGCTTTTCGATGAGCTCGG	CCCGCACGATGAAGAGTAGA
FPS1	TAAACCCGACCCATCGAACG	TTGGTGTCCCTCAATCGCTC
FPS2	TTGCTCATGGCGGGAGAAAA	TGCCAAGTGTCTCAGGATCA
GGPS11	TTGTGAACTCGTCGGAGGTG	TGTGGTTGGTCGGTTTTCCA
**dolichol biosynthesis**		

CPT3	GCGCTTATGTCGATGCTG	CAGACTCAACCTCCTCAGG
LEW1	CATTTGAATGACGCATTAAGAGTTG	AGTTCCCATATGTACTATCTCAGTG
PPRD2	AATCCGAGCCAAGCGAAAGA	TCGCAATCAGTAAACCCGCA
DOK	GGGTTCAATGACCGAGCCTT	ACCGTTTTCTTTCCTGTCTTGC

**MEP pathway**		
DXS	GCGTGCTTATGACCAGGTTG	ATGTGTCGGACCATCAGCTC
DXR	TCCAGGAGAGCAAGGAGTGA	TGCAGCAACCGTAGGCTTTA
MCT	CGGTTGGAGCAGCTGTACTT	TCACCTGTGGTGTCTGCATT
CMK	CCCGAGAAGCATGTTCCACT	ACACACCATTGCTGGTCACA
MDS	AACCAACCAAAACGCTTCCG	TAACACATCGCCATCGGAGT
HDS	TGGCGTTTCGATCGCAATCAT	CGTCATAGCTATCCCACGCT
HDR	CGACTTGTCCTTGGGTGACA	ACGCAGTTGCAATCGTCTCT
**Carotenoid biosynthesis**		
PSY	GCTGCTCTCGCTGATACAGT	GTTCCAGCGACGTAGTAGCA
PDS3	ACCTTCTGAGCGTGTATGCC	ATCCATTCCTCTGCTGGTGC
ZIC1	GATGCATCTCTGGGAGACCG	TGCAGCTACTGTGTTTCCGA
ZDS	TCGAGGGAGTACCTGTGGT	CCAACCGCTCGTTTCAACTG
CRI2	CAAGGAGGTGGGAACACCAA	TCCCCCACGCAGTATAGACC
LYC	ATTTGAGCCGGCCTTATGGG	GGAGTTTGCCTCCTCGTGAA
LUT2	GAGGTGTGTCGAGTCAGGTG	TCCAGAAGCAACAGTGGCAA
