# Supplementary material for: Rab geranylgeranyl transferase activity is required for proper sterol biosynthesis in Arabidopsis thaliana
Source: Plant Cell Physiol. 2025 Dec 10;67(3):346–66. doi: 10.1093/pcp/pcaf166 (PMC13078166; doi:10.1093/pcp/pcaf166)

**Supplemental Figures to manuscript:**

**“ Rab Geranylgeranyl Transferase Activity is Required for Proper Sterol Biosynthesis in *Arabidopsis thaliana.”***

Małgorzata Gutkowska ^1,*,#^, Marta Zajbt-Łuczniewska ^2,*^, Daniel Buszewicz ^2^, Anna Anielska-Mazur ^2^, Agata Lipko ^3^, Cezary Pączkowski ^4^, Grzegorz Spólnik ^5^, Maciej Sojka ^5^, Radosław Jaźwiec ^2^, Emilia Samborowska ^2^, Ewa Swiezewska ^2^, Marta Hoffman ^2^

**Figure S1**

**Structural formulas of the main intermediates and end-products of the MVA, sterol and dolichol pathways.**


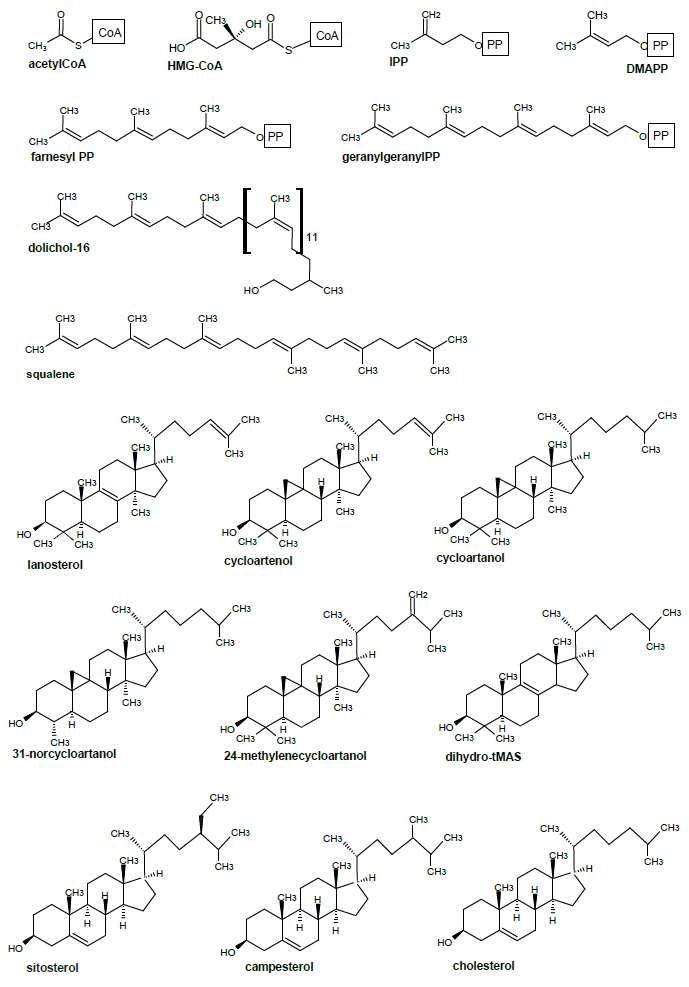


**Figure S2**

**Analysis of 4,4-dimethyl sterols in Arabidopsis WT and *rgtb1-2* plants.**


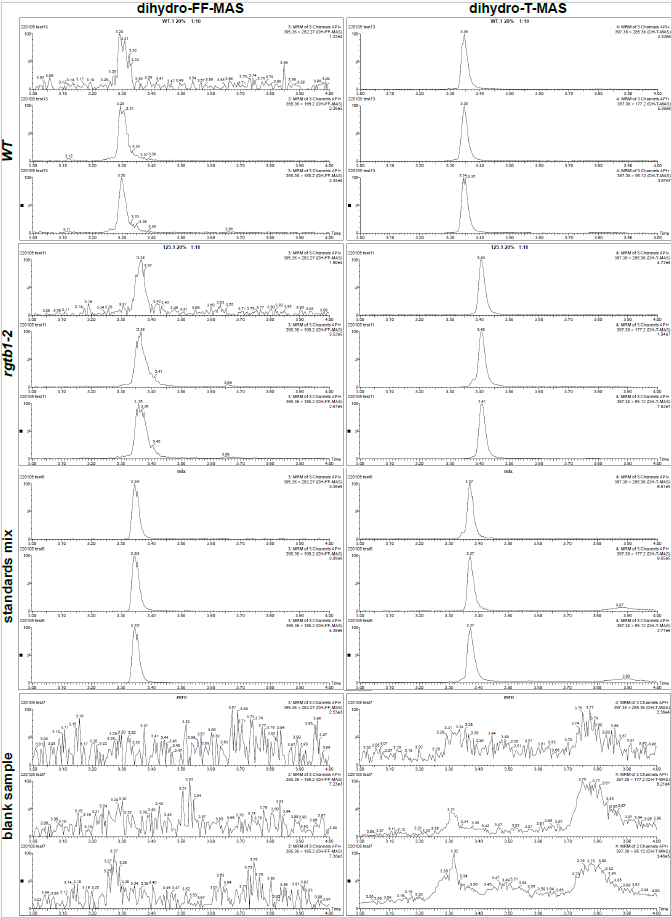


**Fig. S2** Neutral lipids were isolated from whole mature plants grown in soil and fractionated as described in Materials and Methods. Fractions containing sterol precursors were analyzed by UPLC-MS against the external standards of dihydro-t-MAS (4,4-dimethyl-cholesta-8(9)-en-3β-ol ) and dihydro-ff-MAS (4,4-dimethyl-cholesta-8(9), 14-dien-3β-ol). Multiple reaction monitoring (MRM) was applied with concomitant monitoring of ions of *m/z* 285.36; 177.2; 95.12 for t-MAS and *m/z* 282.27; 199.2; 185.2 for dihydro-ff-MAS. In all cases the triple ions peaked at the same retention times.

**Figure S3**

**Analysis of Arabidopsis plants with mutations in the *LAS1* gene.**

**
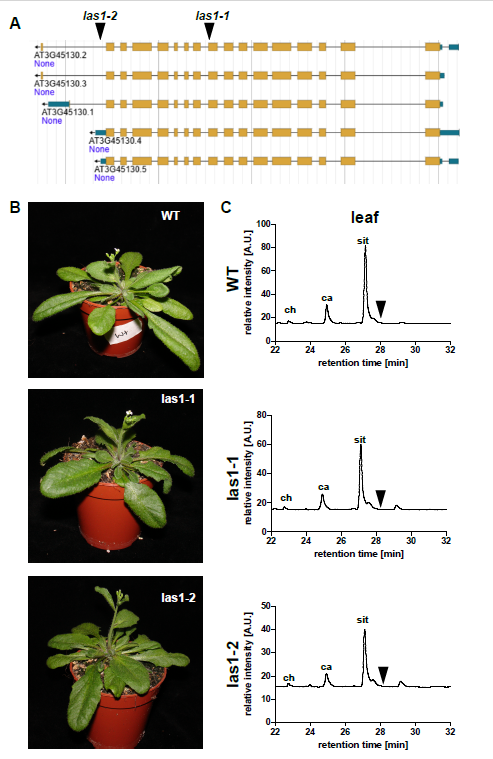
**

**
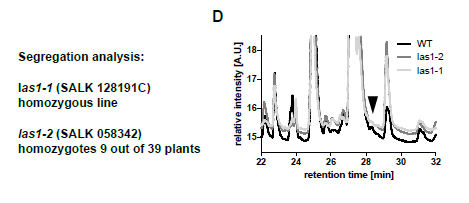
**

**Fig. S3** Phenotypic, chemical and genetic analysis was performed on two T-DNA insertion mutants of the putative Arabidopsis lanosterol synthase gene: *las1-1* and *las1-2*. A) Schematic representation of *LAS1* gene structure. Arrowheads show localizations of T-DNA inserts at the beginning of the 9^th^ intron of the gene for *las1-1* and at the last, 16^th^ intron for *las1-2*. B) Lack of discernible phenotypes of soil-grown 5-week-old *las1-1* and *las1-2* plants in comparison to WT. C) Representative chromatograms showing the pattern of phytosterols extracted from 5-week-old soil-grown WT and *las1-1* and *las1-2* plants. Arrowheads point to the retention time of the external standard of lanosterol. D) Comparison of representative chromatograms as in C), enlarged scale of absorbance axis. Arrowheads point to the retention time of the external standard of lanosterol. E) Genetic analysis of segregation of *las1-1* and *las1-2* homozygous plants. Contingency analysis by Fisher exact test for *las1-2* allele shows Mendelian segregation.

**Figure S4**

**Relative transcription of the genes from the MEP pathway and carotenoid biosynthesis pathway in Arabidopsis *rgtb1* mutants and WT  mature plants.**


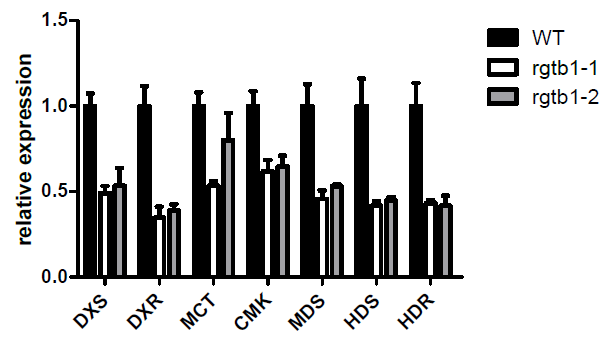


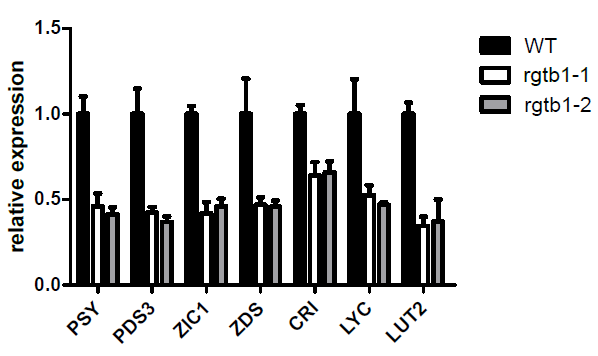


**Fig. S4** Transcription level of the selected genes was analyzed by RT-qPCR, as described in Materials and Methods, in mature *rgtb1* and WT plants grown in soil. Each experiment is a mean of three biological samples, each performed in technical duplicates. Obtained values were normalized to WT values. Graphs represent mean +/- SEM. WT - white bars, *rgtb1-1* - dark grey bars, *rgtb1-2* - light grey bars. Full names of the genes and the corresponding enzymatic activities are given in Table S2.

**Table S1**

**MEP pathway and carotenoid biosynthesis genes in plants**


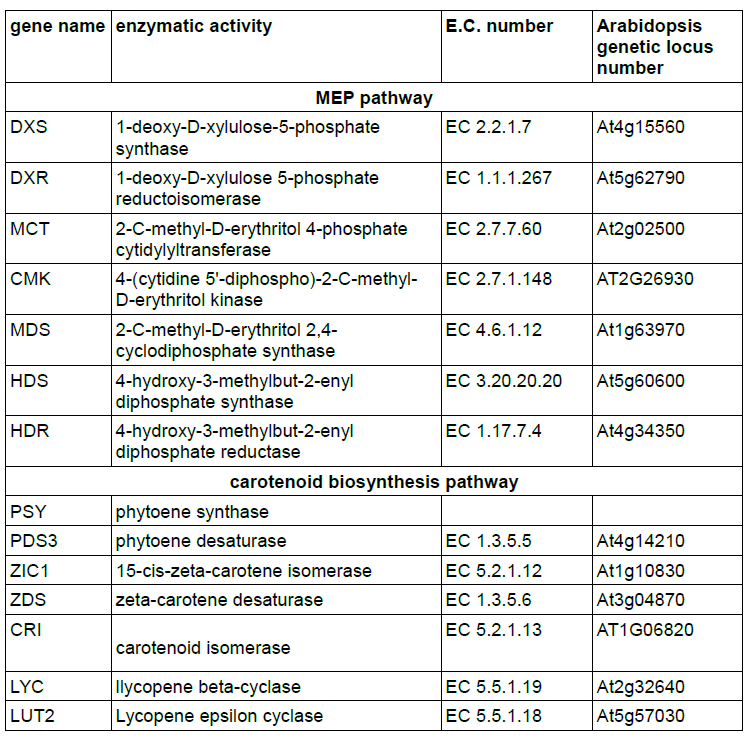


Full names of the genes of the MEP and carotenoid biosynthetic pathways in Arabidopsis, the corresponding enzymatic activities by enzyme classification and genetic loci in Arabidopsis.

**Table S2**

**List of primers used in the study for RT-qPCR analysis of transcription.**


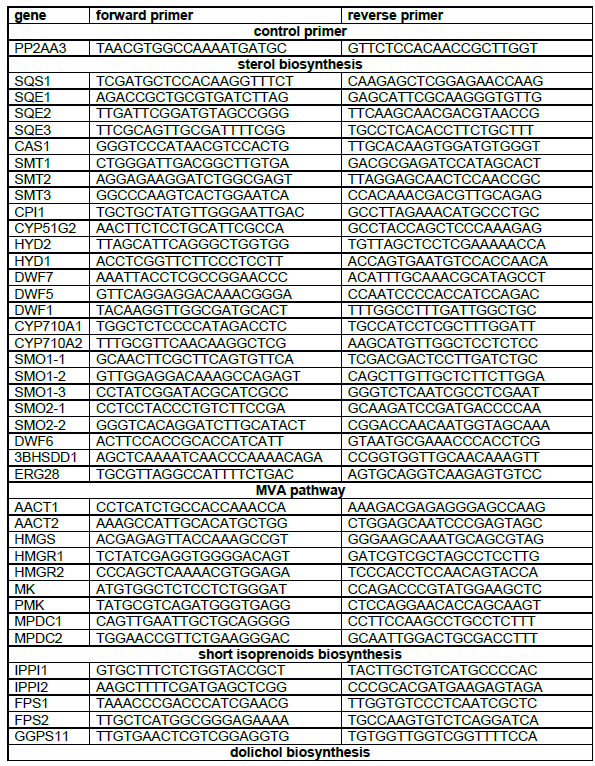

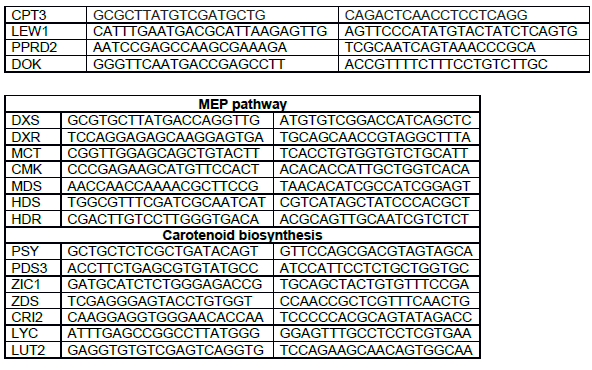

Supplement: Supplementary_materials_pcaf166 [file supplementary_materials_pcaf166.docx]
